# MRNIP condensates promote DNA double-strand break sensing and end resection

**DOI:** 10.1038/s41467-022-30303-w

**Published:** 2022-05-12

**Authors:** Yun-Long Wang, Wan-Wen Zhao, Shao-Mei Bai, Li-Li Feng, Shu-Ying Bie, Li Gong, Fang Wang, Ming-Biao Wei, Wei-Xing Feng, Xiao-Lin Pang, Cao-Litao Qin, Xin-Ke Yin, Ying-Nai Wang, Weihua Zhou, Daniel R. Wahl, Quentin Liu, Ming Chen, Mien-Chie Hung, Xiang-Bo Wan

**Affiliations:** 1 Guangdong Provincial Key Laboratory of Colorectal and Pelvic Floor Diseases, the Sixth Affiliated Hospital, Sun Yat-sen University, Guangzhou, Guangdong 510655 PR China; 2 Department of Radiation Oncology, the Sixth Affiliated Hospital, Sun Yat-sen University, Guangzhou, Guangdong 510655 PR China; 3 Instrumental Analysis Research Center, Sun Yat-sen University, Guangzhou, Guangdong 510275 PR China; 4 Department of Molecular and Cellular Oncology, The University of Texas MD Anderson Cancer Center, Houston, TX 77030 USA; 5 Department of Radiation Oncology, University of Michigan, Ann Arbor, MI 48109 USA; 6 Rogel Cancer Center, University of Michigan, Ann Arbor, MI 48109 USA; 7 Institute of Cancer Stem Cell, Dalian Medical University, Dalian, Liaoning 116044 PR China; 8 State Key Laboratory of Oncology in South China, Cancer Center, Sun Yat-sen University, Guangzhou, Guangdong 510060 PR China; 9 Graduate Institute of Biomedical Sciences and Research Centers for Cancer Biology and Molecular Medicine, China Medical University, Taichung, 404 Taiwan; 10 Department of Biotechnology, Asia University, Taichung, 413 Taiwan

**Keywords:** Double-strand DNA breaks, Radiotherapy, Proteins

## Abstract

The rapid recognition of DNA double-strand breaks (DSBs) by the MRE11/RAD50/NBS1 (MRN) complex is critical for the initiation of DNA damage response and DSB end resection. Here, we show that MRN complex interacting protein (MRNIP) forms liquid-like condensates to promote homologous recombination-mediated DSB repair. The intrinsically disordered region is essential for MRNIP condensate formation. Mechanically, the MRN complex is compartmentalized and concentrated into MRNIP condensates in the nucleus. After DSB formation, MRNIP condensates move to the damaged DNA rapidly to accelerate the binding of DSB by the concentrated MRN complex, therefore inducing the autophosphorylation of ATM and subsequent activation of DNA damage response signaling. Meanwhile, MRNIP condensates-enhanced MRN complex loading further promotes DSB end resection. In addition, data from xenograft models and clinical samples confirm a correlation between MRNIP and radioresistance. Together, these results reveal an important role of MRNIP phase separation in DSB response and the MRN complex-mediated DSB end resection.

## Abstract (web summary)

The MRN complex is a critical sensor and processor of DNA double-strand breaks (DSBs). Here, the authors show that MRNIP forms liquid-like condensates to accelerate the MRN-mediated sensing and end resection of DSB, thereby promoting DSB repair.

## Introduction

DNA double-strand breaks (DSBs) are the most lethal form of DNA damage and threaten genomic stability and cell viability^1,2^. Defects in DSB repair lead to many diseases, such as embryonic death, immunodeficiency, neurological disorders and cancer^3^. Immediately after DSBs are detected, several molecular pathways are activated to arrest cell division and repair the damaged DNA. DSBs are repaired by two major pathways in mammalian cells: homologous recombination (HR) and non-homologous end joining (NHEJ)^4^. HR is an error-free repair pathway requiring the sister chromatid as a recombination template and functions in the S and G2 phases of the cell cycle^5^. By contrast, NHEJ does not require a template and can be applied throughout interphase^6^.

The MRE11/RAD50/NBS1 (MRN) complex is the sensor of DSBs and initiates DNA damage response^1^. This complex localizes to DNA lesions immediately after DSB formation and recruits and induces the autophosphorylation of ataxia telangiectasia mutated (ATM), which further phosphorylates substrates for DNA repair, including the MRN complex^7,8^. Further, the phosphorylated MRN complex initiates DNA end resection and generates 3′-single-stranded DNA for RPA complex and RAD51 loading, which are essential for single strand DNA (ssDNA) stability and sufficient DNA strand exchange of the paired molecules^9^. The MRN complex is a highly conserved protein complex that consist of three subunits: MRE11, RAD50 and Nibrin (NBS1). MRE11 exhibits both 3′ to 5′ exonuclease and endonuclease activities, both of which are essential for DNA end resection^1,10^. RAD50 contains two ATP-binding motifs and exhibits ATPase activity, which are important for the nuclease activity of MRE11 and control the switch of the exonuclease/endonuclease activities of MRE11^11,12^. NBS1 contains two adjacent BRCT domains and functions as an adaptor protein essential for the DNA binding and nuclease activity of the MRN complex^13^. To date, although the regulatory mechanisms underlying the DNA binding and nuclease activities of MRN complex have been well-studied, how the MRN complex senses and binds to DNA rapidly following DSB formation remains unclear.

Recently, liquid-liquid phase separation (LLPS), or condensation, has been implicated as an important mechanism underlying the formation of separated membraneless compartments in cells, such as the nucleolus and paraspeckle^14,15^. When molecules undergo LLPS, they condense into a dense phase, which often form high concentrated liquid droplets; the remaining solution forms a dilute phase^16^. The mechanisms underlying LLPS functions include molecular sponges, reaction crucibles and organizational hubs^17^. Several studies have linked LLPS to DNA damage repair. Damage-induced long noncoding RNAs (dilncRNAs)-driven 53BP1 condensation at damaged DNA lesions is required for DNA repair and p53-dependent gene activation^18,19^. Rad52, a key factor of HR in yeast, is found to create DNA repair centres by the fusion of phase-separated droplets^20^. TopBP1, an activator of ATR, assembles nuclear condensates to switch on ATR signaling^21^. Therefore, LLPS may play a pivotal role in DNA damage repair.

Here, upon performing a screen, we identified MRN complex interacting protein (MRNIP) undergoing liquid-liquid phase separation in vivo and in vitro. MRNIP condensates concentrate the MRN complex into liquid-like droplets in the nucleus. After DSB formation, these MRNIP droplets move to damaged DNA, resulting in rapid binding of damaged DNA to the MRN complex and accelerated ATM activation and DSB end resection, therefore promoting the homologous recombination (HR)-mediated DSB repair. These results uncover MRNIP condensate as an essential regulator of HR-mediated DSB repair.

## Results

### MRNIP forms puncta in tumor cells and its high expression is associated with the radioresistance and poor prognosis of colorectal cancer (CRC) patients

**Fig. 1:**
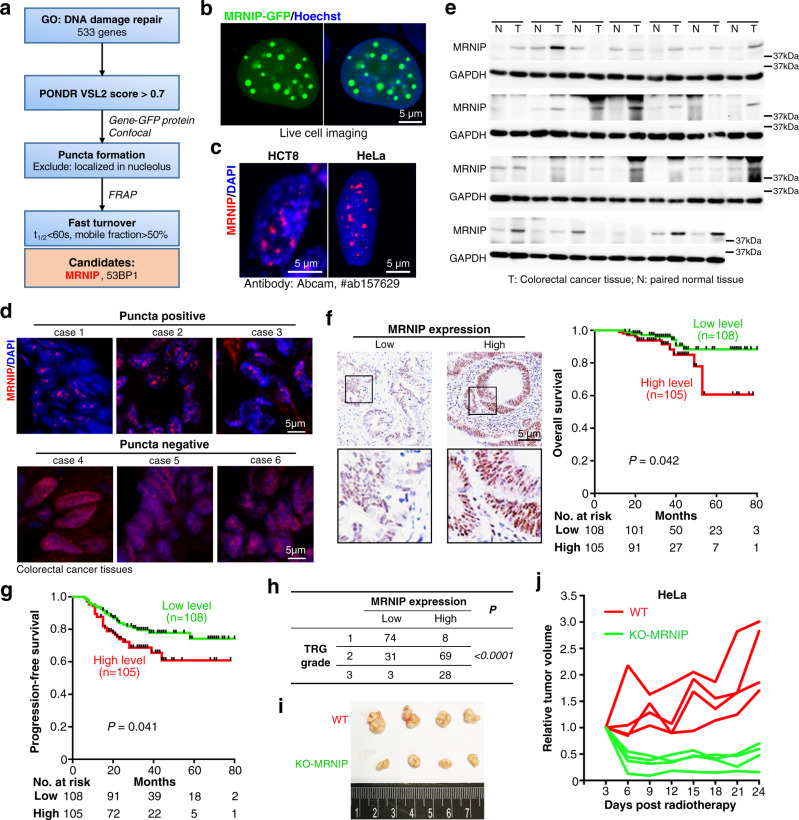
MRNIP formed puncta in tumor cells and its high expression was associated with the radioresistance and poor prognosis of CRC patients. **a** Screening of DNA damage repair-related proteins that undergo LLPS. Details could be found in Supplementary Figure 1a. **b** MRNIP-GFP showed puncta in the nucleus of HEK 293 T cells. HEK 293 T cells were transfected with plasmid for 24 h before observation. **c** Immunofluorescence of endogenous MRNIP in HCT8 and HeLa cells. **d** Immunofluorescence assay showing that MRNIP formed puncta in 3 out of 6 CRC tissues. Frozen sections of CRC tissues were analyzed using Immunofluorescence assay. **e** MRNIP protein was upregulated in CRC tissues. Twenty-seven CRC tissues and adjacent normal tissues were analyzed. **f**, **g** Higher MRNIP expression was correlated with shorter survival time of CRC patients. The correlations were analyzed with Kaplan-Meier curve and Log-rank test. **h** CRC patients with higher MRNIP level were more resistant to radiotherapy. TRG, Tumor regression grade. For (**f**–**h**), CRC tissues from 213 patients were analyzed. **i**, **j** Xenograft model showed that MRNIP depletion sensitized tumor cells to radiation. For (**j**), each line represents one xenograft.

To investigate whether LLPS participates in DNA damage repair, all annotated DNA repair genes in the Gene Ontology database (Supplementary Table 1) were identified and subjected to a screening described in Fig. 1a (detailed in Supplementary Fig. 1a). Finally, MRNIP (also known as c5orf45) and 53BP1 were identified as candidates that exhibit LLPS properties. In agreement with this result, 53BP1 has been reported to form liquid-like condensates to promote p53 induction upon DNA damage^18,19^. Although MRNIP has been found to promote DNA repair^22^, its underlying mechanism remains unknown. Here, we aimed to characterize the condensation of MRNIP and its function in DNA damage repair.

Ectopic expressed MRNIP-GFP or MRNIP-mEGFP protein showed obvious puncta formation in HEK 293T cells (Fig. 1b and Supplementary Fig. 1b-c). Moreover, immunofluorescence assays using three different antibodies showed that endogenous MRNIP also formed puncta in HCT8 and HeLa cells (Fig. 1c and Supplementary Fig. 1d–f), and these puncta were located apart from nucleolus (Supplementary Fig. 1g). Interestingly, MRNIP puncta were also detected in 3 out of 6 colorectal cancer (CRC) tissues (Fig. 1d), and existed in both γ-H2A.X positive and negative tumor cells (Supplementary Fig. 1h). In γ-H2A.X positive cells, the colocalization between MRNIP puncta and γ-H2A.X foci were observed (Supplementary Fig. 1h). Furthermore, compared with paired normal colorectal tissues, MRNIP protein level was increased in CRC tissues (Fig. 1e), and its expression level was not correlated with the level of proliferation marker Ki67 (Supplementary Fig. 2a). Significantly, higher MRNIP level was correlated with the shorter survival time (Fig. 1f, g and Supplementary Fig. 2b–d) and poorer radiotherapy response (Fig. 1h and Supplementary Fig. 2e) of CRC patients. Additionally, MRNIP knockout sensitized tumor to radiotherapy in cellular (Supplementary Fig. 2f) and xenograft model (Fig. 1i, j and Supplementary Fig. 2g).

### MRNIP undergoes liquid-liquid phase separation

**Fig. 2:**
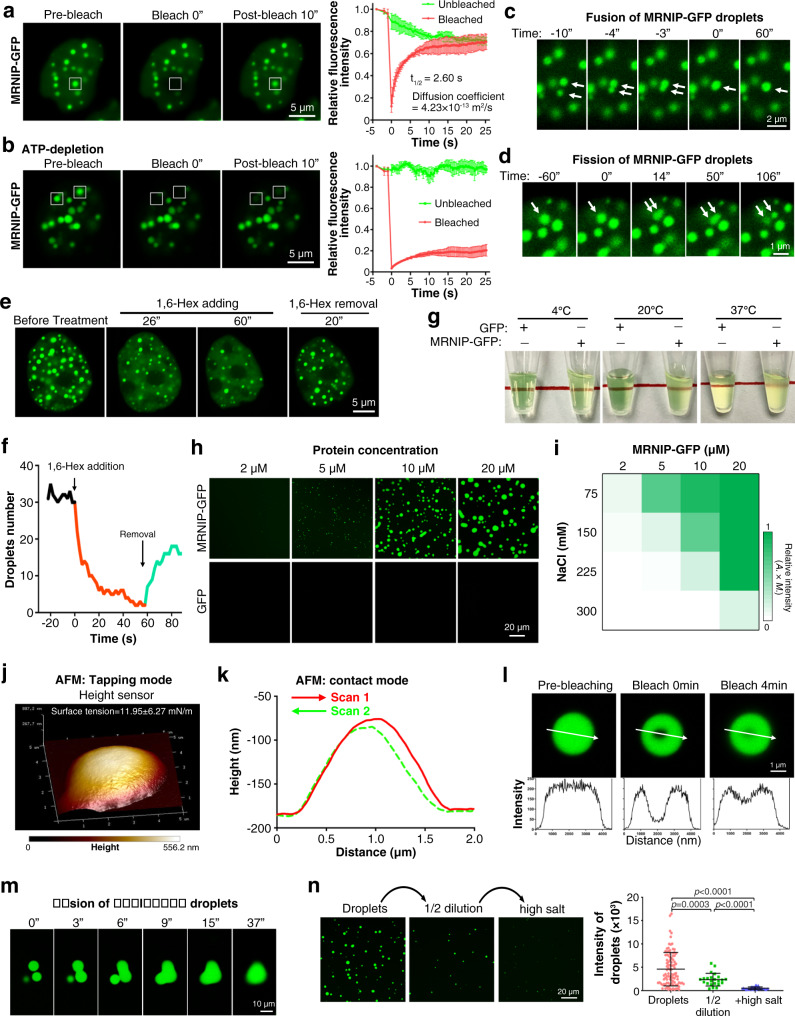
MRNIP undergoes liquid-liquid phase separation. **a** FRAP of MRNIP-GFP puncta in cells. **b** FRAP of MRNIP-GFP puncta in ATP-depleted cells. For (**a, b**), data are presented as means ± SEM; *n* = 3 biological replicates. **c** Fusion of adjacent MRNIP-GFP droplets was observed in cells. **d** One MRNIP-GFP droplet fissured to form two smaller droplets. **e**, **f** MRNIP-GFP droplets were disrupted by 10% 1,6-hexanediol and recovered after removal of 1,6-hexanediol. For (**a**–**f**), HeLa cells were transfected with plasmid for 24 h before observation using confocal microscopy. **g** MRNIP solution was muddied in a temperature-independent manner, whereas the GFP solution remained clear. **h** MRNIP-GFP droplets that formed in buffers containing 150 mM NaCl were observed with confocal microscopy. **i** The impact of protein concentration and NaCl concentration on the formation of MRNIP-GFP droplets. The fluorescence intensity of droplets is presented as the area × mean intensity (*A. × M*.). **j**, **k** Characterization of the morphology of MRNIP droplets using AFM in tapping mode (**j**) or contact mode (**k**). **l** A region within the MRNIP-GFP droplets was photobleached, and fluorescence recovered rapidly. **m** Three in vitro-formed MRNIP-GFP droplets fused to form a larger droplet. **n** MRNIP-GFP droplets were disrupted by dilution and increasing NaCl concentrations. Droplets formed in buffer containing 10 μM MRNIP-GFP and 150 mM NaCl at pH 7.4 and mixed with equal volume of buffer containing 150 mM NaCl at pH 7.4; high salt, 300 mM NaCl. Data are presented as means ± SEM. *n* = 99 (Droplets), *n* = 23 (1/2 dilution), *n* = 40 ( + High salt). Two-tailed unpaired Student’s *t* test.

We next asked whether MRNIP puncta exhibit features of liquid-like condensates. The 3-dimensional images of MRNIP-GFP foci showed that MRNIP-GFP formed droplets in cells (Supplementary Movie 1 and Supplementary Fig. 3a). An essential hallmark of liquid-like condensates is internal dynamic reorganization and rapid exchange kinetics^23^. A fluorescence recovery after photobleaching (FRAP) assay was performed to study the dynamics of MRNIP droplets in live cells. After photobleaching, MRNIP-GFP or MRNIP-mEGFP droplets recovered their fluorescence within 10 s (t_1/2_ = 2.60 s), with a diffusion coefficient of ~0.423 μm^2^/s (Fig. 2a and Supplementary Fig. 3b). However, depletion of adenosine triphosphate (ATP) via glucose deprivation and oligomycin treatment abrogated FRAP of MRNIP-GFP (Fig. 2b), indicating that the rapid exchange of MRNIP between condensates and the dilute phase was an energy-dependent process. Additionally, photobleaching of a region within the MRNIP-GFP droplets was associated with rapid recovery of fluorescence (Supplementary Fig. 3c, d). Importantly, we observed adjacent droplets fusing to form a larger droplet (Fig. 2c, Supplementary Fig. 3e–g and Supplementary Movie 2) as well as a large droplet fissuring into two smaller droplets (Fig. 2d, Supplementary Fig. 3h, i and Supplementary Movie 3), which is consistent with the liquid-like property of MRNIP droplets. Moreover, it was reported that the properties of chromatin could influence the fusion of large liquid domains^24^. We next asked whether chromatin influence the fusion of MRNIP condensates. Our result showed that the fusion of MRNIP droplets was limited by chromatin, but the fusion event had no influence on chromatin property (Supplementary Movie 6). Furthermore, MRNIP droplets were disrupted by 1,6-hexanediol, a compound known to disrupt liquid-like condensates, and recovered rapidly after removing 1,6-hexanediol (Fig. 2e, f and Supplementary Fig. 3j, k). These results indicate that MRNIP forms liquid-like droplets in cells consistent with previously reported LLPS condensates.

We next investigated whether the MRNIP protein formed droplets in vitro. When diluted in buffers containing 150 mM NaCl, purified recombinant MRNIP-GFP protein muddied the solution in a temperature-dependent manner, whereas solutions with purified GFP protein remained clear (Fig. 2g and Supplementary Fig. 4a, b). Observation of the solution under a fluorescence microscope revealed GFP-positive droplets floating in the solution and settling onto a coverslip (Fig. 2h, Supplementary Fig. 4c and Supplementary Movie 4). Phase separation of MRNIP was accelerated by high protein and low salt concentrations but was suppressed by a high salt concentration (Fig. 2i and Supplementary Fig. 4d, e). Consistent with previous reports^25^, mimicking the crowding of the intracellular environment with PEG-8000 significantly enhanced the formation of MRNIP droplets (Supplementary Fig. 4f). Using atomic force microscopy (AFM), MRNIP condensate was characterized as a droplet with a smooth surface (surface tension = 11.95 ± 6.27 mN/m) (Fig. 2j and Supplementary Fig. 4g, h). When analysed with AFM in contact mode, the height curves from the bidirectional scan were slightly shifted, resulting from the liquid-like property of MRNIP droplets (Fig. 2k). Furthermore, the fluorescence of a region within a MRNIP-GFP droplet was diminished by photobleaching and recovered rapidly (Fig. 2l and Supplementary Fig. 4i). Like the cellular observations, adjacent MRNIP-GFP droplets could fuse into a larger droplet (Fig. 2m, Supplementary Fig. 4j, k and Supplementary Movie 5), which further confirmed the liquid-like property of MRNIP droplets. Additionally, dilution of the droplet-containing solution reduced the number and size of droplets, while increasing the salt concentration of the diluted solution further disrupted the droplets (Fig. 2n and Supplementary Fig. 4l), suggesting that highly concentrated MRNIP condensates could be resolved when the physiological conditions change, which distinguish them from gel or solid-like condensates.

### The intrinsically disordered region 1 is required for MRNIP phase separation

**Fig. 3:**
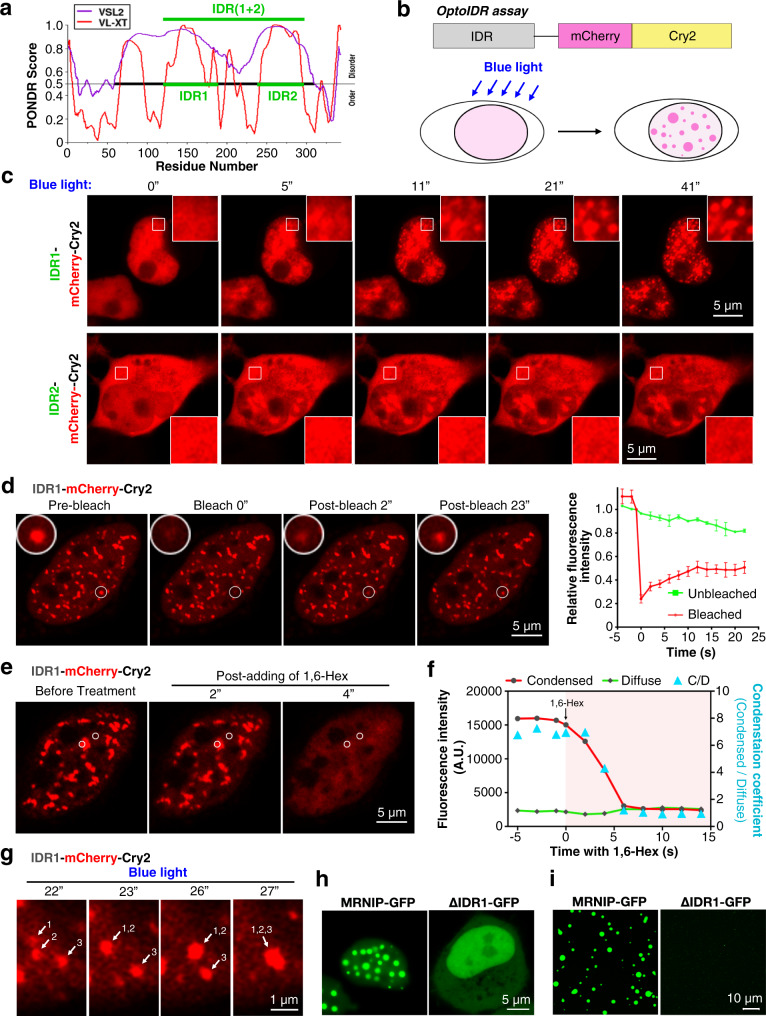
The IDR1 is required for MRNIP phase separation. **a** The disordered region of MRNIP was analysed using PONDR. **b** Schematic of the optoIDR assay. Cells expressing recombinant protein with an IDR, mCherry and Cry2 were exposed to blue light (488 nm). **c** IDR1- or IDR2- Cry2-mCherry was expressed in cells, which were stimulated with blue light to induce condensation. **d** FRAP of blue light-induced IDR1-Cry2-mCherry droplets. *n* = 3 foci analysed in 3 independent experiments. Data are presented as means ± SEM. **e**, **f** Blue light-induced IDR1-Cry2-mCherry droplets were sensitive to 10% 1,6-hexanediol. **g** IDR1-Cry2-mCherry droplets fused to form a larger droplet upon stimulation with blue light. **h**-**i** IDR1-deleted MRNIP could not form liquid-like droplets in vivo (**h**) or in vitro (**i**). The droplets formed in a buffer described in Fig. 2n and a protein concentration of 10 μM was used in (**i**). For (**c**–**h**), HeLa cells were transfected with plasmid for 24 h before observation using confocal microscopy.

Previous studies have implicated the intrinsically disordered region (IDR) of proteins in LLPS^26,27^. We then used the optoIDR assay to examine whether both or any one of two IDRs in MRNIP (Fig. 3a) were required for MRNIP condensation; this assay increases the local concentration of IDR-containing protein and utilizes blue light stimulation to form droplets in vivo (Fig. 3b)^25,28^. Upon blue light stimulation, recombinant proteins containing Cry2-mCherry and full length or IDR1 + IDR2 of MRNIP formed droplets rapidly, whereas those containing only Cry2-mCherry remained diffuse (Supplementary Fig. 5a–c). Fusion of sequences containing IDR1 to Cry2-mCherry also facilitated the rapid formation of droplets upon blue light stimulation, whereas sequences containing IDR2 failed to form droplets (Fig. 3c). Most importantly, blue light-induced droplets showed properties of LLPS, including rapid FRAP, high sensitivity to 1,6-hexanediol and fusion between adjacent droplets (Fig. 3d–g and Supplementary Fig. 5d). In addition, IDR1-deleted MRNIP could not form droplets in vivo or in vitro (Fig. 3h, i and Supplementary Fig. 5e), suggesting that IDR1 is required for MRNIP condensation. Furthermore, we investigate if the functional phosphorylation site at S100/115^22^ of MRNIP would regulate its condensates formation. Here, we found that S100/115A or S100/115E mutation has no impact on the formation of MRNIP condensates (Supplementary Fig. 5f). Although MRNIP has been reported to play roles in HR-mediated DSB repair, which is restricted to S to G2 phases^29^, its condensates were detected in both cyclin A2 positive and negative cells (Supplementary Fig. 5g) and condensates-containing cell population remained unchanged in serum starved and re-stimulated cells (Supplementary Fig. 5h), indicating that cell cycle is not a regulator of MRNIP phase separation.

### MRNIP condensates incorporate the MRN complex and relocalize to damaged DNA

**Fig. 4:**
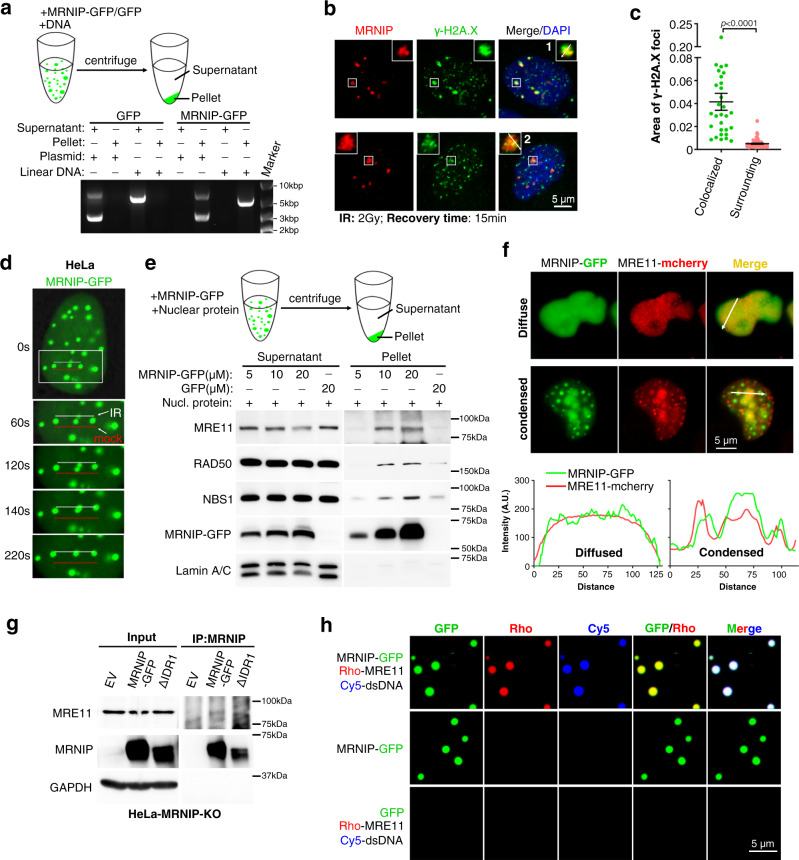
MRNIP condensates incorporate the MRN complex and relocalize to damaged DNA. **a** MRNIP-GFP droplets compartmentalized DNA from solutions. MRNIP-GFP droplets that formed in vitro were incubated with DNA solutions and fractionated by centrifugation. **b** γ-H2A.X localized within MRNIP droplets in cells after 15 min recovery post irradiation. MRNIP-GFP-expressing HeLa cells were irradiated (2 Gy) and subjected to γ-H2A.X detection at indicated time. **c** The quantification of γ-H2A.X foci area in (**b**). Colocalized, γ-H2A.X foci colocalized with MRNIP puncta; Surrounding, γ-H2A.X foci didn’t colocalize with MRNIP puncta. Data are presented as means ± SEM. Colocalized, *n* = 30; Surrounding, *n* = 61. Two-tailed unpaired Student’s *t* test. **d** MRNIP-GFP condensates moved to the microirradiation-induced DNA damage site. IR, microirradiation; mock, no IR. HeLa cells were transfected with plasmid and incubated with 10 μM BrdU for 24 h before microirradiation. **e** MRNIP-GFP droplets compartmentalized the MRN complex from the nuclear extract of HeLa cells. The indicated protein was incubated with 10 µg of HeLa nuclear extract in buffer containing 150 mM NaCl (pH 7.4) for 20 min at 20 °C and fractionated by centrifugation. **f** MRNIP droplets incorporated with MRE11 in cells. Live-cell image of cells co-expressing MRE11-mCherry and MRNIP-GFP. HeLa cells were transfected with indicated plasmids for 24 h before observation. **g** Co-immunoprecipitation assay showed that IDR1-deleted MRNIP did not interact with MRN complex. **h** MRNIP-GFP droplets incorporated MRE11 and dsDNA in vitro. The indicated proteins or dsDNA were incubated in buffer containing 150 mM NaCl, pH 7.4, for 20 min at 20 °C. Rho Rhodamine.

Phase separation-formed condensates are known to recruit factors and provide separate space for biological processes. We hypothesized that MRNIP condensates may enhance the binding between MRN complex and damaged DNA to accelerate their interaction. To test whether MRNIP droplets incorporate DNA in vitro, pre-formed MRNIP droplets were incubated with linearized or circular plasmid DNA and separated by centrifugation. Surprisingly, all DNA was incorporated into the MRNIP droplets in vitro (Fig. 4a and Supplementary Fig. 6a). Additionally, the mixture of fluorescent-tagged DNA and MRNIP droplets was analysed with a confocal microscope, and their colocalization was observed (Supplementary Fig. 6b). Interestingly, when the mixture was separated by native polyacrylamide gel electrophoresis (PAGE), DNA was retained in the sample well due to the presence of the MRNIP droplets; adding PEG-8000 to enhance MRNIP droplet formation remarkably increased the amount of DNA retained in the well (Supplementary Fig. 6c). Most importantly, the localization of γ-H2A.X within MRNIP condensates was observed in cells 15 min after irradiation (Fig. 4b, c, Supplementary Fig. 6d, e). Interestingly, two different modalities were detected: several individual small γ-H2A.X foci were presented in a large MRNIP punctum; a big γ-H2A.X punctum merged with a MRNIP punctum (Fig. 4b, Supplementary Fig. 6e). To examine whether MRNIP condensates relocalized to or just formed a new condensate at DNA damage site, we performed microirradiation assay. As a control, RNF168 could be recruited onto DNA damage site and form high concentrated puncta (Supplementary Fig. 6f), whereas MRNIP condensates moved to DNA damage site, instead of forming new puncta (Fig. 4d). These results suggest that MRNIP droplets could be relocalized to and incorporate damaged DNA lesion.

As a key partner of MRNIP, the MRN complex was detected in pelleted MRNIP droplets incubated with nuclear extracts in vitro (Fig. 4e and Supplementary Fig. 6g, h). In cells, endogenous or ectopically expressed MRE11 was colocalized with MRNIP-GFP condensates in the nucleus but not colocalized in cells with diffuse MRNIP (Fig. 4f). IF assay showed that endogenous MRNIP puncta were colocalized with MRE11 in 12.59 ± 1.84% of HeLa cell (Supplementary Fig. 6i). In addition, CoIP assay showed that MRE11 was interacted with wildtype MRNIP, but not with IDR1-deleted MRNIP, which had no LLPS capacity (Fig. 4g), suggesting that their interaction was dependent on the LLPS of MRNIP. Furthermore, both MRE11 and dsDNA were recruited into MRNIP droplets in vitro (Fig. 4h). These results indicate that MRNIP condensates may incorporate the MRN complex and move to damaged DNA.

### MRNIP condensates accelerate the MRN complex loading and DNA damage response

**Fig. 5:**
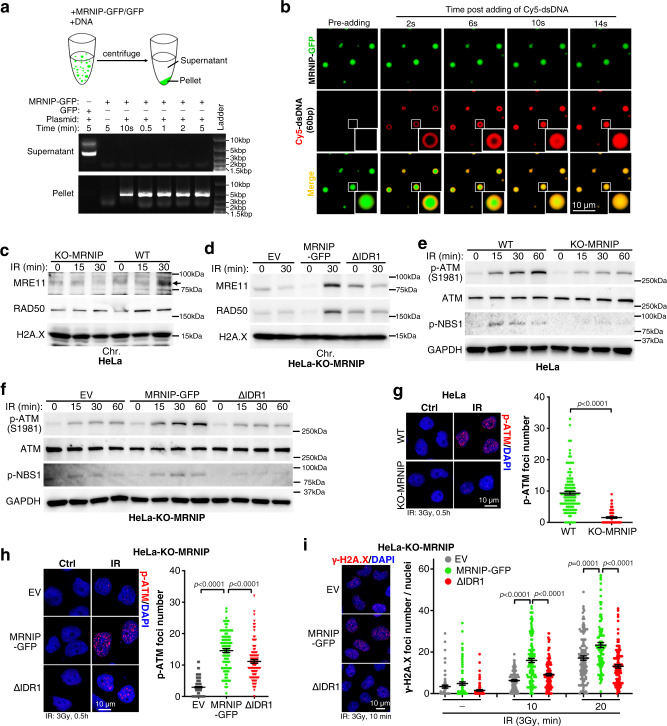
MRNIP condensates accelerate the MRN complex loading and DNA damage response. **a**, **b** MRNIP condensates incorporated DNA from solutions within 10 s. Ten micromolar MRNIP was incubated with 15 ng/µL plasmid DNA or 0.5 µM Cy5-dsDNA in buffer containing 150 mM NaCl, pH 7.4. **c** MRNIP depletion reduced radiation-induced MRN complex binding to chromatin. **d** The impact of wild-type or IDR1-deleted MRNIP on the radiation-induced binding of the MRN complex to DNA. For (**c**, **d**), chromatin was fractionated at the indicated time after irradiation (10 Gy). EV, empty vector; ΔIDR1, MRNIP-ΔIDR1-GFP. **e**, **f** The impact of MRNIP condensates on radiation-induced ATM and NBS1 phosphorylation. IR: 3 Gy. **g**, **h** IF assays were performed to analyse the impact of MRNIP on radiation-induced ATM phosphorylation. HeLa cells were irradiated with 3 Gy. Data are presented as means ± SEM. Two-tailed unpaired Student’s *t* test. **g** *n* = 116 (WT), *n* = 77 (KO-MRNIP). **h** *n* = 107 (EV), *n* = 93 (MRNIP-GFP), *n* = 103 (ΔIDR1). **i** IF assays were performed to analyze the impact of MRNIP on radiation-induced γ-H2A.X phosphorylation. HeLa cells were exposed to 3 Gy X-ray. Data are presented as means ± SEM. IR^-^: *n* = 69 (EV), *n* = 87 (MRNIP-GFP), *n* = 87 (ΔIDR1); IR 10 min: *n* = 124 (EV), *n* = 122 (MRNIP-GFP), *n* = 119 (ΔIDR1); IR 20 min: *n* = 111 (EV), *n* = 96 (MRNIP-GFP), *n* = 112 (ΔIDR1). Two-tailed unpaired Student’s *t* test. For (**c**–**i**), HeLa-KO-MRNIP cells stably expressing sgRNA-resistant MRNIP-GFP, MRNIP-ΔIDR1-GFP (ΔIDR1) and empty vector (EV) were used.

As a sensor of DSBs, the MRN complex binds to DNA damage lesions rapidly after DSB formation. Interestingly, MRNIP droplets could quickly sequester all DNA in solution, as shown, within 10 s after the DNA was introduced (Fig. 5a, b and Supplementary Fig. 7a). Theoretically, this rapid and strong recruitment of DNA by MRNIP condensates may facilitate the loading of the MRN complex to damaged DNA. In line with our hypothesis, after radiation-induced DNA damage, 1,6-hexanediol treatment significantly reduced the binding between the MRN complex and genomic DNA, which was similar to the outcome of MRNIP depletion (Fig. 5c and Supplementary Fig. 7b–d). Moreover, re-expressing MRNIP in MRNIP-depleted cells increased the binding of the MRN complex with DNA, whereas re-expressing the IDR1-deleted mutant of MRNIP had no impact (Fig. 5d and Supplementary Fig. 7e, f). Additionally, the examination of radiation-induced MRE11 foci also showed a similar result (Supplementary Fig. 7g, h), indicating that MRNIP condensation is essential for the loading of the MRN complex.

After binding to DSBs, the MRN complex recruits and activates ATM, a kinase that coordinates DNA repair by phosphorylating other proteins, to initiate the DNA damage response^3,30^. Consistently, MRNIP depletion and 1,6-hexanediol treatment inhibited radiation-induced ATM autophosphorylation at S1981 (Fig. 5e, g and Supplementary Fig. 7i–j) but had no influence on p-CHK1-S345 level, a downstream substrate of ATR (Supplementary Fig. 7k), whereas re-expression of MRNIP in MRNIP-depleted cells rescued p-ATM levels (Fig. 5f, h). However, re-expression of MRNIP-ΔIDR1 in MRNIP-depleted cells failed to rescue this phenotype (Fig. 5f, h). Interestingly, 1,6-hexanediol treatment had no impact on radiation-induced p-ATM in MRNIP-KO HeLa cells (Supplementary Fig. 7l), suggesting that MRNIP mediated the suppression of p-ATM by 1,6-hexanediol. Consistent with the activation of ATM, MRNIP condensates accelerated the radiation-induced γ-H2A.X accumulation (Fig. 5i and Supplementary Fig. 7m). Together, these results indicate that MRNIP condensates enhance MRN complex loading after DSB formation and accelerate the ATM-mediated DNA damage response.

### MRNIP phase separation enhances DNA end resection

**Fig. 6:**
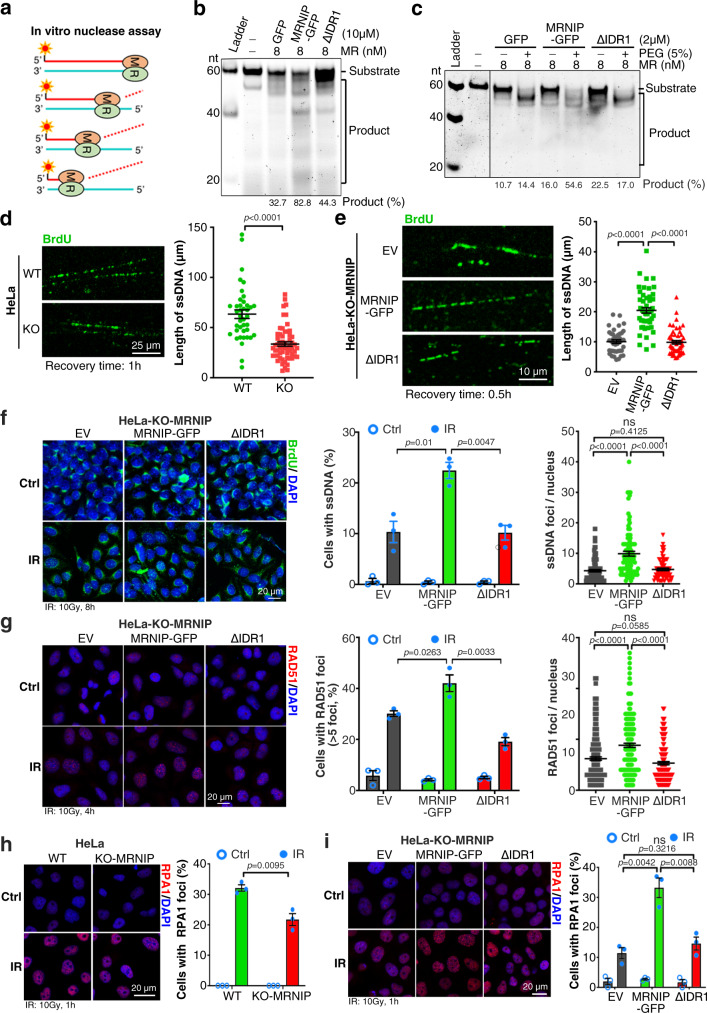
MRNIP condensation promotes DNA end resection. **a**, **b** MRNIP condensates promoted MRE11/RAD50-mediated dsDNA degradation in vitro. **c** The impact of PEG-8000 on MRNIP participated degradation of dsDNA by MRE11/RAD50 complex. For (**b**, **c**), the final concentration of GFP, MRNIP-GFP or ΔIDR1-GFP was 10 μM (**b**) and 2 μM (**c**). **d**, **e** ssDNA fiber length was examined via IF assay with anti-BrdU antibody. Data are presented as means ± SEM. Two-tailed unpaired Student’s *t* test. **d**, *n* = 40 (WT), *n* = 59 (KO-MRNIP). **e** *n* = 41 (EV), *n* = 52 (MRNIP-GFP), *n* = 50 (ΔIDR1). **f** In situ detection of ssDNA in cells confirmed that MRNIP condensates promote radiation-induced ssDNA formation. Data are presented as means ± SEM. Two-tailed unpaired Student’s *t* test. *Middle panel*: *n* = 3 biological replicates; *Right panel*: *n* = 91 (EV), *n* = 95 (MRNIP-GFP), *n* = 78 (ΔIDR1). **g** Restoration of MRNIP in HeLa-MRNIP-KO cells increased RAD51 foci. Data are presented as means ± SEM. Two-tailed unpaired Student’s *t* test. *Middle panel*: *n* = 3 biological replicates; *Right panel*: *n* = 205 (EV), *n* = 211 (MRNIP-GFP), *n* = 167 (ΔIDR1). **h** MRNIP knockout reduced radiation-induced RPA1 foci. Data are presented as means ± SEM; *n* = 3 biological replicates. Two-tailed unpaired Student’s *t* test. **i** Restoration of MRNIP in HeLa-MRNIP-KO cells increased RPA1 foci. Data are presented as means ± SEM, *n* = 3 biological replicates. Two-tailed unpaired Student’s *t* test. For (**d**–**i**), cells were treated with 10 Gy X-rays and recovered for 8 h (**f**), 4 h (**g**) or 1 h (**h**, **i**) before analysis. For (**e**–**g**, **i**), HeLa-KO-MRNIP cells stably expressing sgRNA-resistant MRNIP-GFP, MRNIP-ΔIDR1-GFP (ΔIDR1) and empty vector (EV) were used. ns, no significance.

Next, we examined whether MRNIP condensates promoted the MRN complex-mediated DNA end resection. Purified key player MRE11/RAD50 (MR) complex efficiently catalysed the 3′ to 5′ exonucleolytic degradation of dsDNA in vitro (Fig. 6a and Supplementary Fig. 8a, b), and MRNIP-GFP significantly enhanced the exonucleolytic digestion of dsRNA substrates by MR complex, whereas the IDR1-deleted mutant had no impact (Fig. 6b). Most importantly, a low concentration of MRNIP-GFP, that could not form condensates, had no obviously impact on MR activity (Fig. 6c). As shown in Supplementary Fig. 4f, the crowding agent PEG-8000 could induce the condensation of MRNIP at its lower concentration. We interestingly found that, although PEG-8000 significantly enhanced MR activity in the absence of MRNIP, MRNIP further accelerated PEG-8000-enhanced dsDNA degradation (Fig. 6c and Supplementary Fig. 8c).

Furthermore, the impact of MRNIP condensates on DNA end resection was investigated in vivo. Anti-BrdU antibody could only detect BrdU-labelled ssDNA, therefore denaturation of genomic DNA was normally required for BrdU incorporation assay. If the denaturation process was skipped, the endogenous ssDNA derived from DNA end resection could be detected. After cells were irradiated, cellular genomic DNA was spread on a glass slide and subjected to an immunofluorescence assay with anti-BrdU antibody. Consistent with the results from the in vitro assay, depletion of MRNIP restricted the extension of ssDNA (Fig. 6d), while restoration of MRNIP had the opposite effect (Fig. 6e). However, restoration of IDR1-deleted mutants of MRNIP had no impact on the length of ssDNA (Fig. 6e). Furthermore, ssDNA derived from DNA end resection was detected in situ. MRNIP depletion reduced the ratio of ssDNA-containing cells, in which the ssDNA content was also decreased (Supplementary Fig. 8d, e). By contrast, re-expressing MRNIP increased the ratio of ssDNA-containing cells, as well as the ssDNA content per nucleus, whereas deletion of IDR1 abolished these effects (Fig. 6f). In addition, the detections of ssDNA-binding protein RAD51 and RPA1 foci showed similar results (Fig. 6g–i and Supplementary Fig. 8f). These data suggest that MRNIP accelerates DNA resection in an LLPS-dependent manner.

### MRNIP phase separation promotes DNA damage repair and radioresistance

**Fig. 7:**
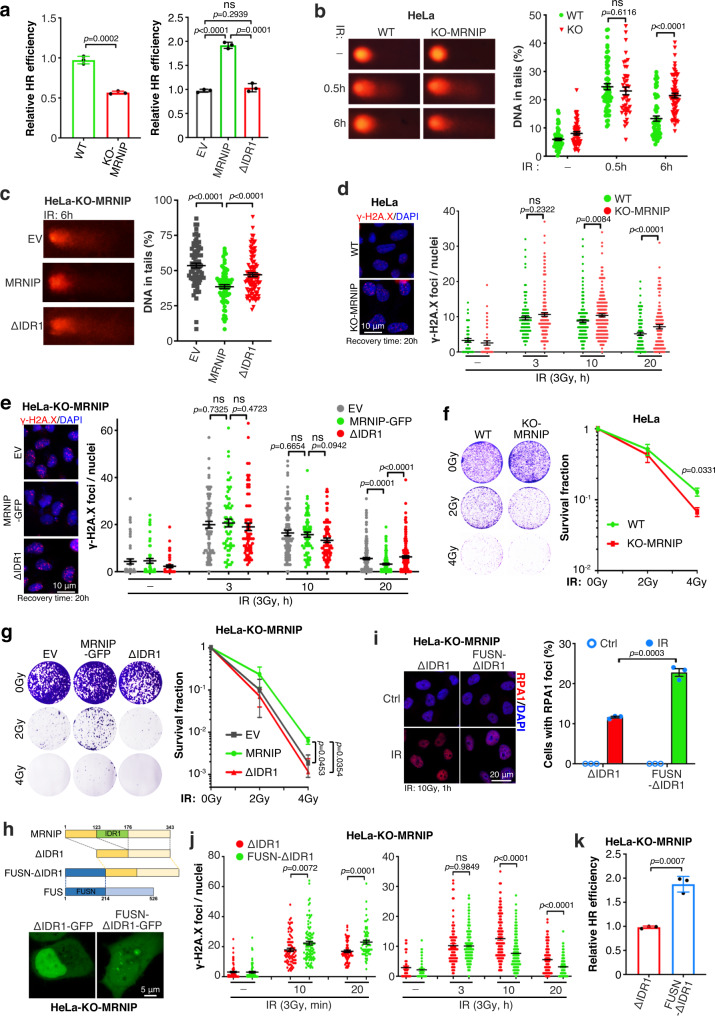
MRNIP phase separation enhances DNA repair and radioresistance. **a** The influence of MRNIP phase separation on HR-mediated DNA repair was explored with an DSB repair reporter. Data are presented as means ± SEM; *n* = 3 biological replicates. **b**, **c** Comet assays were conducted to explore the impact of MRNIP depletion on DNA repair. Data are presented as means ± SEM. **b** *n* = 58, 52, 62, 45, 59, 69 (from left to right column). **c** *n* = 76, 71, 98 (from left to right column). **d**, **e** γ-H2A.X was detected by immunofluorescence assay, and the γ-H2A.X content was counted. Data are presented as means ± SEM. **d** *n* = 48, 51, 96, 182, 164, 199, 103, 116 (from left to right column). **e** *n* = 50, 50, 50, 75, 63, 73, 81, 62, 77, 190, 141, 173 (from left to right column). **f**, **g** Colony formation assay showed that MRNIP-enhanced tumor radioresistance was ascribed to its LLPS capacity. Data are presented as means ± SEM; *n* = 3 biological replicates. For (**a**, **c**, **e**, **g**), HeLa-KO-MRNIP cells stably expressing sgRNA-resistant MRNIP-GFP, MRNIP-ΔIDR1-GFP (ΔIDR1) and empty vector (EV) were used. **h** IDR of FUS restored the LLPS capacity of MRNIP-ΔIDR1. HeLa-KO-MRNIP cells were transfected with plasmid for 24 h before observation. **i** FUSN-ΔIDR1 increased RPA1 foci after radiation in HeLa-MRNIP-KO cells. Data are presented as means ± SEM; *n* = 3 biological replicates. **j** FUSN-ΔIDR1 accelerated radiation-induced γ-H2A.X accumulation and DNA repair. Data are presented as means ± SEM. *Left panel*: *n* = 96, 79, 79, 102, 64, 63 (from left to right column). *Right panel: n* = 48, 45, 92, 128, 163, 176, 126, 131 (from left to right column). **k** The impact of FUSN-ΔIDR1 on HR was analyzed using DSB repair reporter. Data are presented as means ± SEM; *n* = 3 biological replicates. For (**h**–**k**), HeLa-KO-MRNIP cells stably expressing sgRNA-resistant MRNIP-ΔIDR1-GFP (ΔIDR1) and FUSN-MRNIP-ΔIDR1-GFP (FUSN-ΔIDR1) were used. All *P* values were calculated using two-tailed unpaired Student’s *t* test. ns, no significance.

DNA end resection is a key step in HR and HR-mediated DSB repair. Consistent with the essential role of MRNIP in DNA end resection, MRNIP knockout reduced the efficiency of HR-mediated DSB repair, and re-expressing of MRNIP-ΔIDR1 mutant could not rescue it. (Fig. 7a). Cell cycle analysis showed that MRNIP had no influence on cell cycle (Supplementary Fig. 8g-h), indicating a direct regulation between MRNIP and HR. Furthermore, a comet assay and γ-H2A.X detection showed that MRNIP depletion inhibited radiation-induced DNA damage repair (Fig. 7b, d), and restoration of MRNIP reversed this effect (Fig. 7c, e). Most importantly, cells expressing the IDR1-deleted mutant of MRNIP, which did not form condensates, had a DNA repair capacity similar to that of MRNIP-KO cells (Fig. 7c, e), indicating that LLPS of MRNIP promoted the repair of radiation-induced DSBs. Enhanced DNA repair capacity is a main cause of tumor resistance to radiotherapy. Consistently, MRNIP depletion sensitized tumor cells to radiotherapy in an in vitro colony formation assay (Fig. 7f) and re-expressing MRNIP in MRNIP-KO cells enhanced the radioresistance of tumor cells, whereas this effect was diminished by deleting IDR1 (Fig. 7g).

To further confirm the role of phase separation in MRNIP function, the IDR of FUS (amino acids 1-214 at the N-terminal, FUSN), a well-characterized RNA binding protein with LLPS capacity, was inserted to the N-terminal of MRNIP-ΔIDR1 (designated as FUSN-ΔIDR1) to restore its phase separation capacity (Fig. 7h). Interestingly, compared with MRNIP-ΔIDR1, re-expressing of FUSN-ΔIDR1 in HeLa-KO-MRNIP cells significantly promoted radiation-induced ATM activation, RPA1/RAD51 foci formation and the accumulation of γ-H2A.X (Figs. 7i, j–*left panel* and Supplementary Fig. 8i, j). Consistently, γ-H2A.X detection after recovery and DSB repair reporter assay showed that FUSN-ΔIDR1 enhanced HR-mediated DSB repair (Fig. 7j–*right panel*, k).

## Discussion

**Fig. 8:**
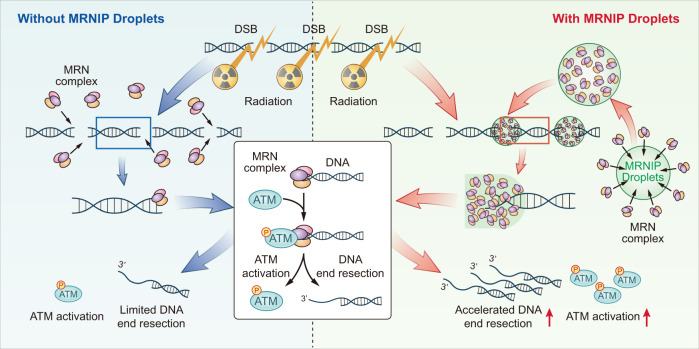
Model of MRNIP condensates function in DNA damage sensing and end resection. MRNIP condensates concentrate the MRN complex into liquid-like droplets. After DSB formation, these MRNIP droplets move to and incorporate damaged DNA, resulting in rapid binding of DNA to the MRN complex and accelerated ATM activation and DNA end resection.

In this study, we characterized MRNIP forming highly concentrated condensates in vitro and in vivo. MRNIP condensates concentrate the MRN complex. Upon DSBs formation, MRNIP condensates move to and incorporate DSBs, which are quickly bound to the concentrated MRN complex, resulting in an accelerated DNA damage response and end resection (Fig. 8).

After DSB formation, a series of DNA repair factors are recruited and concentrated at damaged DNA lesions to form DNA repair centers^31^. These factors form highly concentrated foci^32,33^, which are usually observed in protein LLPS or condensation^34,35^. Therefore, it is reasonable to propose that some of these factors are likely to be phase separated. To verify this hypothesis, we conducted a screen and identified that MRNIP and 53BP1 had the potential to form phase-separated condensates in cells. Interestingly, when we were studying these factors, the LLPS of 53BP1 and its function in DNA repair were reported by two other individual groups^18,19^, indicating that our screen is sufficient to discover potential phase-separated DNA repair factors. Sinan Kilic et al. reported that 53BP1 formed phase-separated condensates at the DNA damage site, which concentrates tumor suppressor protein p53 and promotes the 53BP1-dependent induction of p53 and p53 target gene expression^19^. Furthermore, dilncRNAs drive molecular crowding of 53BP1 into foci that exhibit LLPS condensate properties to promote DNA repair^18^. In addition, the accumulation of 53BP1 at damaged DNA lesions was impaired by poly(ADP-ribose) (PAR)-seeded liquid demixing of intrinsically disordered proteins, such as FUS or TAF15^36^. In this study, we showed that MRNIP phase separation-derived condensates accelerated the recognition of damaged DNA by the MRN complex and the process of DNA end resection. Interestingly, Camilla Frattini et al recently reported that TOPBP1 amplified ATR/Chk1 signaling via a very similar mechanism^21^. Furthermore, we did not detect a close association between MRNIP and 53BP1 condensates (Supplementary Fig. 8k), suggesting that MRNIP may be a specific regulator of HR-mediated DSB repair. These findings, coupled with previous reports, indicate that phase separation is incorporated in different stages of the DNA damage response, including the sensing of damaged DNA, activation and amplification of DNA damage response signaling, and DNA repair processes.

The recognition of damaged DNA is an initial step in the DNA damage response^37^. The MRN complex and Ku70/80 heterodimer are sensors of DSBs in HR- and NHEJ-mediated DNA repair pathways, respectively^38^. The MRN complex recruits the protein kinase ATM to damaged DNA, induces its autophosphorylation and activates its protein kinase activity^39^. Meanwhile, hundreds of ATM substrates involved in the DNA damage response have been defined and applied in chromatin remodelling, cell cycle regulation and DNA repair progression^40^. Thus, the recognition of damaged DNA by the MRN complex is essential for HR-mediated DNA repair. Recently, it has been reported that UBQLN4 removes ubiquitylated MRE11 from damaged chromatin, thereby repressing HR and promoting NHEJ for DSB repair^41^. C1QBP competitively binds with MRE11/RAD50 to form the MRC complex, which limits the binding of MRE11 to DNA and inhibits its nuclease activity in normal cells. When DSBs occur, MRE11 is phosphorylated by ATM and dissociates from the MRC complex, consequently binding to NBS1 to form the MRN complex and further anchoring to DNA^11^. However, the regulation of MRN complex-mediated DSB sensing process is poorly understood. Here, we characterized MRNIP condensate as an accelerator in the sensing of DSBs by MRN complexes. MRNIP phase-separated condensates concentrate the MRN complex in the nucleus. After DSB formation, MRNIP droplets moved to damaged DNA lesion, thereby provide a niche containing highly concentrated MRN complex, resulting in accelerated MRN-mediated DSB sensing and rapid activation of ATM. Meanwhile, we noticed the limitation of our study that the impact of MRNIP condensates on DSB sensing was concluded from the in vitro assay and p-ATM analysis. We didn’t capture the MRN-mediated DSB sensing process directly, which should be happening within a very short time (seconds to minutes). Though it was not characterized in the present study, effort is being made to disclose the underlying mechanism and the answer of this point will be addressed in our future work.

The initiation stage of DSB end resection is catalysed by the MRN complex and is a tightly regulated process^42,43^. Recently, many factors have been reported to regulate DNA end resection by influencing the DNA binding capacity^44^, nuclease activity^45^, protein expression^46^ or assembly of the MRN complex^11^. Our results from the in vitro and in vivo experiments show that MRNIP condensates promote DSB end resection by enhancing the loading of MRN complex. Especially, restoration the LLPS capacity of IDR-deleted MRNIP using FUS N-terminal rescued the function of MRNIP in DNA end resection, indicating the requirement of LLPS for MRNIP function.

Aberrant DNA repair is one of the main causes of tumor radioresistance^47,48^. Consistently, dysregulation of the MRN complex has been implicated in the radioresistance of many cancer types^13^. For CRC, high expression of the MRN complex is correlated with poor prognosis in both postoperative and neoadjuvant radiotherapy-treated patients^49^. Overexpression of the MRN complex enhances the radioresistance of CRC, and targeting RAD50 sensitizes CRC cells to radiotherapy^50,51^. In agreement with these observations on the MRN complex, our results reveal that high MRNIP expression correlates with poor response to neoadjuvant radiotherapy in CRC patients. Additionally, knocking out MRNIP or abolishing the phase separation capacity of MRNIP via IDR1 deletion sensitized tumor cells to radiotherapy. Most importantly, MRNIP puncta were also observed in both γ-H2A.X positive and negative tumor cells of CRC tissues. However, whether these MRNIP puncta observing in CRC tissues are formed by phase separation or just binding to damaged chromatin needs further investigation. Furthermore, the analysis of radiation proctitis tissues showed that comparing with the normal rectum tissues or tissues distal from proctitis, MRNIP expression was reduced in radiation proctitis tissues (Supplementary Fig. 9), indicating that the reduction of MRNIP may sensitize rectum cells to radiation and lead to the development of radiation proctitis.

Taken together, MRNIP condensates may serve as a surrogate for radioresistant cancer patients, and targeting MRNIP condensates may be a potential strategy for sensitizing tumor cells to radiotherapy.

## Methods

### Cell lines and tissues

HEK 293T and HeLa cells were grown in Dulbecco’s modified Eagle’s medium (DMEM, Gibco, ThermoFisher Scientific, Waltham, Massachusetts, USA) supplemented with 2 mM L-glutamine, 100 units/mL penicillin-streptomycin (15140122, HyClone, South Logan, UT, USA) and 10% foetal bovine serum (FBS, Gibco) and cultured at 37 °C with 5% CO_2_. For routine cell culture passaging, trypsin-EDTA (25300062, Gibco) was used to detach cells from the cell culture flask or plates.

MRNIP knocked out HeLa cells were constructed using CRISPR/cas9. Briefly, HeLa cells were infected with lentivirus containing MRNIP-targeting sgRNA for 48 h before selected with 2.5 μg/ml puromycin for 3 days. After the verification of MRNIP knockout with Western blotting, cells were designated as HeLa-KO-MRNIP. Cells infected with lentivirus derived from lentiCRISPRv2 empty vector were used as the wildtype control.

Human CRC and adjacent non-tumor rectal tissues, radiation proctitis tissues, normal rectum tissues and tissues distal from proctitis were obtained from the Tissue Bank of the Sixth Affiliated Hospital, Sun Yat-sen University. All patients underwent radical CRC surgery, and both tumor and normal adjacent tissues were confirmed histologically. The American Association of Cancer/College of American Pathologists (AJCC/CAP) tumor regression grading (TRG) system was employed to stratify the treatment response according to the volume of residual tumor cells. Informed consent was obtained from each patient, and the protocol was approved by the Institutional Research Ethics Committee of the Sixth Affiliated Hospital of Sun Yat-sen University.

### Construction of plasmids

Human cDNA was cloned into pCDH-CMV-MCS-EF1-Puro, pGEX-6P-1 or a modified version of the pcDNA3.0 vector^52^. The base vectors were engineered to include a C-terminal EGFP, mEGFP, mCherry or mCherry-CRY2 followed by a stop codon. Encoding cDNA sequences generated by PCR were inserted in-frame before EGFP, mEGFP, mCherry or mCherry-CRY2. The human proteins used in this paper were as follows: full-length MRNIP (WT), amino acids 1-343; MRNIP-IDR1, amino acids 123-176; MRNIP-IDR2, amino acids 242-295; MRNIP-IDR (1 + 2), amino acids 123-295; MRNIP-ΔIDR1, amino acids 1-343 with the IDR1 domain (amino acids 123-176) removed; FUSN-MRNIP-ΔIDR1: N-terminal of FUS (amino acids 1-214) + MRNIP-ΔIDR1; full-length MRE11: amino acids 1-708; full-length NPM1: amino acids 1-294; full-length FBL (Fibrillarin): amino acids 1-321; and full-length RNF168: amino acids 1-571. To generate MRNIP-KO cell lines, a lentiCRISPRv2 vector was used to create a plasmid targeting the MRNIP genomic locus with the following sgRNA sequence: TGCCAGTGAAGAAGAAAAC.

### Live-cell imaging

Cells were seeded in 35 mm glass-bottom dishes twelve hours before transfection with the plasmid. Twenty-four hours after transfection, Hoechst 33342 (4082, Cell Signaling Technology, CST, Danvers, MA, USA) was added to the medium at a final concentration of 1 μg/mL, and the cells were incubated for 10 min at 37 °C. Hoechst-containing medium was replaced with fresh complete medium before the cells underwent live-cell imaging using a Zeiss LSM880 confocal microscope equipped with an incubation chamber to provide a humidified atmosphere at 37 °C with 5% CO_2_. 1,6-Hexanediol treatment: Cells cultured on 35 mm glass-bottom dishes with complete medium were imaged every 2 s. After the 10th acquisition, cells were incubated with complete medium containing 10% 1,6-hexanediol. After the cells were incubated for 60 s, the culture medium was replaced with complete medium, and the cells were cultured for another 60 s. ATP depletion: Complete DMEM was replaced with glucose-free DMEM (11966025, Gibco), and the cells were cultured for 2 h. 2-Deoxy-D-glucose (HY-13966, MedChemExpress, MCE, Monmouth Junction, NJ, USA) and oligomycin (495455, Sigma-Aldrich, St. Louis, Missouri, USA) was added to the medium at final concentrations of 5 mM and 126 nM, respectively, after which the cells were cultured for another two hours before observation.

### Fluorescence recovery after photobleaching

FRAP was performed on an LSM880 Zeiss microscope with 488 or 561 nm laser. Bleaching was performed at 100% laser power, and images were collected every 0.25 s. Images were further processed, and the fluorescence intensity was calculated using ZEN3.1 (Blue Edition). The background intensity was subtracted, and values were measured relative to the prebleaching time points. For kinetic analysis, relative fluorescence intensity was plotted against time by setting the intensity before bleaching as 1.0 and fitted to an equation as follow: (1) FI = *C*_*0*_(1-exp(*k*(*t*))). In this equation, *C*_*0*_ is the maximum recovery at *t* = infinity, *t* is time in seconds and *k* is a constant which can be calculated by the equation. The *t*_1/2_ value was calculated by ln (2)/*k*. Diffusion coefficient was analysed and calculated via Fick’s law of Diffusion as described previously^53^. Briefly, a droplet was bleached with a certain beam size and required certain time for recovery to reach half that of the outside, then the diffusion coefficient *D* was calculated as follow: (2) $D=\frac{1}{{t}_{1/2}}{\left(\frac{r}{2{{{{{{\rm{Erfc}}}}}}}^{-1}1/2}\right)}^{2}$, in which Erfc^-1^(0.5)$\,\sim$ 0.4769 and *r* is radius of the beam.

### OptoIDR assay

Cells were transfected with MRNIP-IDR-mCherry-CRY2 plasmids using Lipofectamine 2000 transfection reagent (11668019, Invitrogen) 24 h before imaging. Droplet formation was induced with light pulses at 488 nm (blue light, 50% laser power) every 2 s during the imaging process, and images were captured every 2 s. The FRAP and 1,6-hexanediol treatment assays were performed after stimulation with blue light for 60 s, and images were captured every 2 s in the absence of blue light. Images visible under a Zeiss LSM880 confocal microscope with a 64x oil objective were captured.

### Fraction of nuclear and chromosomal extracts

After irradiation (10 Gy) or 1,6-hexanediol (1.5%) treatment, cells were scraped and resuspended in cytolysis buffer (10 mM HEPES-NaOH, pH = 7.9; 10 mM KCl; 1.5 mM MgCl_2_; and 0.5 mM beta-mercaptoethanol) supplemented with protease inhibitor (04693132001, Roche, Basel, Switzerland) and phosphatase inhibitor cocktail (B15001, Bimake, Shanghai, China), followed by incubation on ice for 20 min. NP-40 was added to a final concentration of 0.2%, and cells were vortexed and kept on ice for 2 min before they were centrifuged at 16,000 × g for 15 min. The supernatant was saved as the cytoplasmic protein fraction. The pellet was washed twice with ice-cold 1 × PBS, resuspended in nuclear lysis buffer (10 mM Tris-HCl, pH = 7.6; 420 mM NaCl; 0.5% NP-40; 1 mM DTT; 1 mM PMSF; and 2 mM MgCl_2_) supplemented with protease inhibitor and phosphatase inhibitor cocktail, incubated on ice for 20 min and then centrifuged at 16,000 × g for 15 min. The resulting supernatant was saved as the nuclear protein fraction. The pellet was washed three times with ice-cold 1 × PBS, dispersed in 250 mM HCl with tips and then kept at 4 °C overnight. Approximately 12 h later, the chromosomal extracts were obtained by centrifugation at 16,000 × g for 15 min. Finally, 2.5 M NaOH was added to neutralize the solution.

### Western blotting

Whole-cell extracts were obtained in RIPA buffer (25 mM Tris-HCl, pH = 7.4; 150 mM NaCl; 1% NP-40; 0.5% Na-deoxycholate) supplemented with protease inhibitor and phosphatase inhibitor cocktail. Chromosomal extracts and droplet fractions were prepared as previously described. The primary antibodies used in this paper targeted the following proteins: MRE11 (4847S, CST, 1:1000), Rad50 (3427S, CST, 1:1000), NBS1 (14956S, CST, 1:1000), p-NBS1 (3001S, CST, 1:1000), MRNIP (ab150917, Abcam, Cambridge, MA, USA, 1:1000), Lamin A/C (ab108595, Abcam, 1:4000), p-H2A.X (9718S, CST, 1:1000), GAPDH (60004-1-Ig, Proteintech, Rosemont, IL, USA, 1:5000), p-ATM-S1981 (AP0008, Abclonal, Wuhan, China, 1:1000), p-CHK1-S345 (2348S, CST, 1:1000), α-Tubulin (66031-1-Ig, 1:5000) and H2A.X (A11361, Abclonal, 1:1000). Images were captured with a ChemiDoc imaging system (Bio-rad). Blots images were processed using Image Lab software. The uncropped blots images were provided in the Supplementary Information.

### Immunofluorescence

Cells grown on coverslips were fixed with 4% paraformaldehyde (DF0135, Leagene, Beijing, China) for 15 min at RT, washed three times with 1 × PBS, blocked in blocking buffer (5% goat serum, 0.3% Triton X-100 in 1 × PBS) for at least one hour at RT, and incubated with primary antibodies diluted in blocking buffer for 2 h at RT or 4 °C overnight. After three washes in 1 × PBS, the samples were treated with secondary antibodies tagged with Alexa Fluor 488, 555 or 647 (4408S, 4413S or 4414S, CST) for one hour at RT in the dark. Cells were washed twice in PBS and then stained with DAPI (D9542, Sigma-Aldrich). Glass slides were mounted in ProLong™ Diamond Antifade Mountant (P36965, Invitrogen). Images were acquired using an LSM880 Zeiss confocal microscope and processed by ZEN software (Blue edition). The primary antibodies used in this paper targeted the following proteins: MRNIP (ab157629, Abcam, 1:200; ab150917, Abcam, 1:200; TA330650, Origene, 1:200), MRE11 (ab214, Abcam, 1:100), p-H2A.X (9718S, CST, 1:500), p-H2A.X (80312S, CST, 1:500), p-ATM-S1891 (AP0008, Abclonal, 1:300), RPA1 (2267S, CST, 1:50), TP53BP1 (4937, CST, 1:200) and Rad51 (ET1705-96, HuaBio, Hangzhou, China, 1:100).

### Immunohistochemistry

Formalin-fixed paraffin-embedded specimens were sliced into 5 μm sections and mounted on polylysine-coated slides. After deparaffinization in xylene twice for 10 min and rehydration through a graded series of ethanol, the sections were heated in a microwave for 25 min in antigen retrieval solution (10 μM citrate buffer, pH = 6.0), incubated with 0.3% hydrogen peroxide for 10 min in the dark to block endogenous peroxidase activity and treated with anti-MRNIP antibody (for CRC tissues: ab157629, Abcam, 1:100; for mouse xenograft tissues: TA330650, Origene, 1:100) or anti-Ki67 antibody (ET1609-34, HuaAn biotechnology, Hangzhou, China, 1:200) at 4 °C overnight. Next, the sections were immunostained with a Biotin-Streptavidin HRP Detection System (SP-9000, ZSGB-BIO, Beijing, China) at 37 °C for 35 min before they were subsequently stained with a DAB Detection Kit (ZLI-9018, ZSGB-BIO) for 1 minute, followed by haematoxylin staining (Zymed Laboratories, South San Francisco, CA, USA). After a final series of washes, slides were dried and mounted.

### In situ ssDNA detection by BrdU

Cells were cultured with 10 μM BrdU for 24 h before irradiation (10 Gy). Eight hours after irradiation, cells were subjected to the BrdU assay as described previously^52^ without the denaturation of genomic DNA. Briefly, cells were permeabilized with fixing solution (three volumes of 50 mM glycine solution pH = 2.0 plus seven volumes of absolute ethanol) at 4 °C overnight before they were treated with trypsin solution (0.05% trypsin and 0.05% CaCl_2_ in PBS) at 37 °C for 7 min. After three washes in PBS, cells were subjected to an immunofluorescence assay with anti-BrdU antibody (66241-1-Ig, Proteintech, 1:300).

### Single molecule analysis of resection tracks

Cells were treated with 10 µM BrdU in culture medium for 24 h before irradiation. After the indicated recovery time, cells were spotted on Silane Prep slides (Sigma-Aldrich) and lysed with lysis buffer (200 mM Tris-HCl, pH = 7.4; 50 mM EDTA and 0.5% SDS). Genomic DNA was spread on glass, air-dried and fixed with 3:1 methanol/acetic acid at −20 °C for 15 min, before it was incubated with 70% ethanol at 4 °C for 1 h. Slides were then subjected to an immunofluorescence assay with anti-BrdU antibody (66241-1-Ig, Proteintech, 1:300) and imaged with a Zeiss LSM880 microscope using Airyscan mode.

### Expression and purification of MRNIP-GFP

MRNIP-GFP proteins were expressed in the *Escherichia coli* strain BL21(DE3). Cultures were grown at 37 °C until the OD_600_ reached 0.6–0.7. Then, IPTG was added to a final concentration of 0.1 mM, and cultures were grown at 16 °C for 12–14 h. After centrifugation, cells were collected and resuspended in 1 × PBS supplemented with 1 mM PMSF, lysed by sonication and then centrifuged twice at 15,000 × g for 15 min. The supernatant was applied to GST-tagged purification resin (P2253, Beyotime, Shanghai, China) at 4 °C for 3 h, followed by three washes with 1 × PBS. Excision of the GST tag was performed at 4 °C overnight using human rhinovirus type 14 3 C protease (P2303, Beyotime). The eluted protein was concentrated in high salt buffer (50 mM Tris-HCl, pH = 7.5; 1 M NaCl) and then stored at −80 °C. All purification steps were performed on ice or at 4 °C.

### In vitro droplet assay

In vitro droplet assays were performed to investigate MRNIP droplet formation behaviour in response to changes in PEG8000, pH, salt or protein concentrations. For changes in PEG8000, MRNIP-GFP protein was diluted to the indicated concentration in buffers containing 20 mM Tris-HCl (pH = 7.4) and 150 mM NaCl either with or without 5% PEG8000. For changes in the salt and protein concentration, MRNIP-GFP recombinant protein was diluted to the indicated concentration in 20 mM Tris-HCl (pH = 7.4) containing NaCl at the indicated concentration. For changes in pH and the protein concentration, the indicated concentration of MRNIP-GFP protein was added to 20 mM Tris-HCl of the indicated pH containing 150 mM NaCl. For changes in pH and the salt concentration, 10 μM MRNIP-GFP protein was added to 20 mM Tris-HCl of the indicated pH containing NaCl at the indicated concentration. Droplet assays were performed in a reaction volume of 20 µL in PCR tubes mixed by pipetting and vortexing. The reaction mixtures were incubated at 20 °C for 10 min and then pipetted onto glass slides. Images were captured using a Zeiss LSM880 confocal microscope with a 64× oil objective and further processed by ZEN software (Blue edition, 3.1). Fluorescence intensity was measured by Image J.

### Atomic force microscope imaging

AFM images were captured in tapping mode using icon scanner AFM instrument (Dimension FastScan Bio, Bruker, Germany) equipped with a high-resonance microscope. Parameters of the cantilever we used were as follows: length, 70 μm; width, 10 μm; thickness, 0.6 μm; frequency, 150 kHz; spring constant, 0.35-1.4 N/m (Scanasyst-Fluid + , Bruker). Microscope was used to observe and select MRNIP-GFP droplets for nanoscale imaging with AFM. AFM imaging conditions were as follows: scan size, 5.00 × 5.00 μm^2^; scan rate, 0.501 Hz; pixel size, 20 × 20 pixels. All imaging was performed at room temperature. NanoScope Analysis software (Version 1.40, Bruker Corporation) was used to process the images. Surface tension was calculated using the following formula:^54^ (3) $\sigma=\frac{{F}_{{{{{{\rm{ret}}}}}}}}{2\pi r}=\frac{{F}_{{{{{{\rm{pull}}}}}}-{{{{{\rm{in}}}}}}}}{2\pi r{{{{{\rm{cos }}}}}}\left({\theta }_{e}\right)}$, where *F*_ret_ is the retention force, *F*_pull-in_ is the pull-in force, *r* is the radius of the nanoneedle, and *θ*_*e*_ is the equilibrium contact angle between the meniscus and nanoneedle.

### Droplet pelleting

Nuclear proteins of HeLa cells were obtained as described above. Linearized pCDH-puro plasmid DNA was obtained by digestion with BamHI. The indicated concentrations of MRNIP-GFP or GFP recombinant proteins were incubated with 1 μg/μL nuclear protein or 15 ng/µL DNA (circular or linearized plasmid) at 20 °C for 20 min in buffer containing 20 mM Tris-HCl (pH = 7.4) and 150 mM NaCl, followed by centrifugation at 10,000 × g and 4 °C for 10 min. The supernatant was retained, and the pellet was dissolved in SDS sample buffer (50 mM Tris, pH = 6.8; 2% SDS; 10 mM EDTA; 10% glycerol; 0.1% bromophenol blue) for immunoblotting or in high-salt TE buffer (20 mM Tris-HCl, pH = 8.0, 1 mM EDTA, 1 M NaCl) for DNA electrophoresis.

### Expression and purification of MRE11 in insect cells

To generate baculovirus, pQB3-MRE11 plasmid and qBac-III (qBac^®^ Bacmid) were co-transfected into sf9 insect cells with Promega FuGENE HD transfection reagent (E2311, Promega, Madison, WI, USA) followed by incubation in SFX-insect medium (SH30278.02, Hyclone) supplemented with 2% FBS at 28 °C for 4 days. Medium was collected as P1 baculovirus generation and then subjected to infect sf9 cells for multiple rounds to generate high titer baculovirus. Sf9 cells were infected with high titer baculovirus and incubated in shake flask for another four to five days. sf9 cells were collected via centrifugation, resuspended in nondenaturing lysis buffer (P2229S, Beyotime) supplemented with 1 mM PMSF, lysed by sonication and then centrifugated twice at 15,000 × g for 15 min. The supernatant was applied to the following purification steps with His-tag protein purification kit (P2229S, Beyotime) according to the manufactural protocol. The eluted protein was concentrated in the high salt buffer (50 mM Tris-HCl, 1 M NaCl, pH = 7.5) and then stored at −80 °C. All the purification steps were performed on ice or at 4 °C.

### Nuclease reactions

Nuclease reactions were performed to evaluate the impact of MRNIP condensates on MRE11/RAD50 exonuclease activity. The MRE11/RAD50 complex was purified as described previously^11^. DNA oligonucleotides labelled with Cy5 at the 5′-end were annealed with unlabelled antisense DNA oligos (1:2) to produce dsDNA (60 bp). The indicated concentration of MRE11/RAD50 was mixed with 5 μM MRNIP-GFP in reaction buffer (25 mM Tris-HCl, pH = 7.5; 2 mM MnCl_2_; 1 mM DTT; 100 mg/ml BSA; 100 mM KCl), followed by the addition of 5 nM Cy5-labelled dsDNA. After a 30 min incubation at 30 °C, the reaction mixtures were separated by denaturing urea PAGE, and gels were scanned with Typhoon 5 (Amersham).

### Flow cytometry analysis

For DNA repair reporter analysis, cells were seeded in 10 cm dish for 24 h before transfected with 2.5 ug pLCN DSB Repair Reporter, 4 ug pCAGGS DRR mCherry Donor EF1a BFP and 2.5 ug pCBASceI plasmid (these plasmids were gifts from Jan Karlseder (Addgene plasmid # 98896; http://n2t.net/addgene:98896; RRID: Addgene_98896)^55^. Forty-eight hours after transfection, cells were trypsinized and resuspended in 1×PBS supplemented with 5% FBS and subjected to flow cytometry analysis (CytoFLEX, Beckman, Pasadena, USA). BFP^+^ cells were gated for mCherry analysis.

For cell cycle analysis, cells were trypsinized, resuspended in a detergent-containing hypotonic solution and subjected to flow cytometry analysis as described previously^52^. Data were analysed using FlowJo X software.

### Comet assay

A comet assay was performed to detect DNA damage by measuring tail DNA. After the indicated recovery time after radiation (10 Gy), cells were detached from 12-well plates with trypsin and resuspended in 1 × PBS. Approximately 10,000 cells were mixed with 1% low-melting agarose (A4018, Sigma-Aldrich) at 37 °C and layered on a slide coated with normal melting agarose. The slide was further coated with 1% low-melting agarose as the top layer. The prepared slides were placed into cold, freshly made lysis solution (10 mM Tris-HCl, pH = 10.0; 2.5 M NaCl; 100 mM EDTA; 1% Triton X-100; 10% DMSO) overnight at 4 °C, followed by three washes in 10 mM Tris-HCl (pH = 10.0) for 5 min to remove detergent. Then, the slides were placed in electrophoresis buffer (300 mM NaOH, 1 mM EDTA, pH > 13.0) for 20 min before electrophoresis at 25 volts and 300 milliamperes for 40 min. After the slides were neutralized in neutralization buffer (0.4 M Tris-HCl, pH = 7.5), they were stained with GelRed (BS354B, Biosharp, Shanghai, China) and imaged with a fluorescence microscope (IX73, Olympus).

### Mouse xenograft models

Two million HeLa or HeLa-KO-MRNIP cells in 100 μL of 1 × PBS/Matrigel (1:1, vol:vol) (356234, Corning, NY, USA) were injected subcutaneously into the right or left posterior flank of 5-week-old male NOG mice (NOD.Cg-*Prkdc*^*scid*^*Il2rg*^*tm1Sug*^/JicCrl) (Charles River, Beijing, China). Two months later, the xenografts were irradiated with a single 5 Gy dose of X-rays. After irradiation, tumors were measured for length (L) and width (W) with callipers every three days until the 24^th^ day after irradiation. Tumor volume was calculated with the following formula: (4) Tumor volume = (L × W^2^) × 0.5. All mice were maintained in ambient room temperature (23 + /−3 °C) with humidity of 40–70% and light/dark cycle of 12 h/12 h. All animal experiments were approved by the Institutional Animal Care and Use Committee of the Sixth Affiliated Hospital of Sun Yat-sen University (Accreditation No. IACUC-2020052503). Experimental procedures were performed in accordance with the Guide for the Care and Use of Laboratory Animals (National Institutes of Health Publication No. 80-23, revised 1996) and according to the institutional ethical guidelines for animal experiments.

### Statistics and reproducibility

Experiments from clinical samples, including Fig. 1d and Supplementary Fig. 1h, were repeated once. Experiments including Figs. 1b, c, 2c–e, h, m, 3c, g–i, 4d–h, 5b–f, 6b, c and Supplementary Figs. 1b–g, 3a, 5a–c, 6a–d, 6f–i, 7b–f, i, k, l, 8a–c, k were repeated independently three times. Statistical analyses for each experiment are indicated in the respective figure legends. Kaplan-Meier survival curves, Log-rank test and Cox proportional hazard regression analyses were performed using SPSS version 13.0 (SPSS Inc., Chicago, IL) to identify prognostic factors. All statistical tests were two-sided and were performed using GraphPad Prism 8 (GraphPad Software Inc., San Diego, CA).

### Reporting summary

Further information on research design is available in the Nature Research Reporting Summary linked to this article.

## Supplementary information

Supplementary Information

Description of Additional Supplementary Information

Supplementary Movie 1

Supplementary Movie 2

Supplementary Movie 3

Supplementary Movie 4

Supplementary Movie 5

Supplementary Movie 6

Reporting Summary

## Data Availability

All data generated or analysed during this study are included in this published article (and its supplementary information files). Source data are provided with this paper.

## Source data

Source Data
