# Supplementary material for: MRNIP condensates promote DNA double-strand break sensing and end resection
Source: Nat Commun. 2022 May 12;13:2638. doi: 10.1038/s41467-022-30303-w (PMC9098523; doi:10.1038/s41467-022-30303-w)
Supplement: Supplementary file 1 — Supplementary Information [file 41467_2022_30303_MOESM1_ESM.pdf]

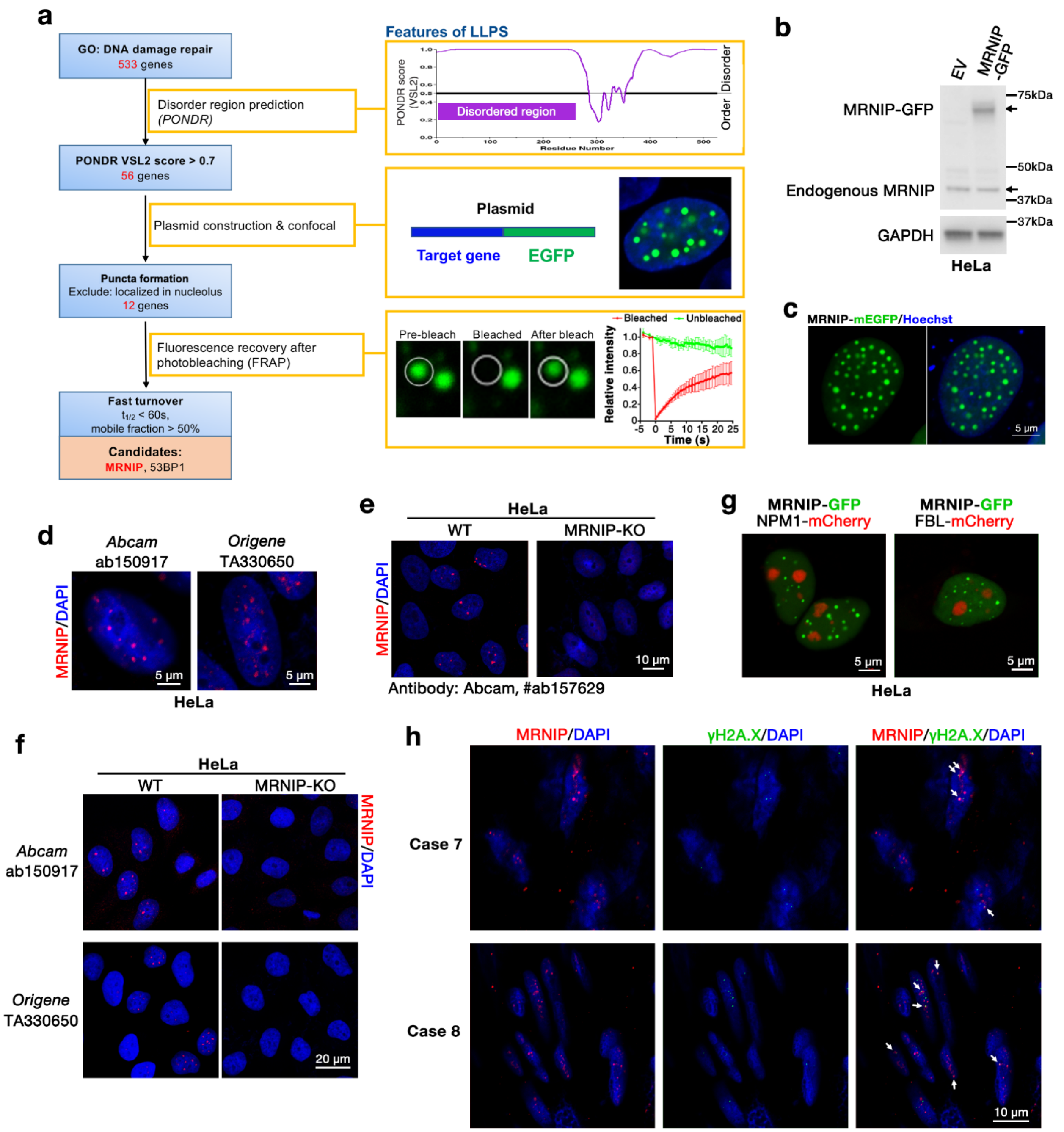

Supplementary Figure 1. MRNIP formed puncta in tumor cells. a Screening of DNA damage repair-related proteins that undergo LLPS. Briefly, all genes annotated as DNA repair genes in the Gene Ontology database were identified and subjected to disorder region analysis with PONDR (http://www.pondr.com/). Fifty-six highly scored proteins were cloned into GFP fusion vectors, which were transduced into cells subjected to living cell imaging in order to detect puncta formation. A total of 16 proteins were found to form puncta in cells. Excluding 4

nucleoli-localizing proteins, 12 puncta-positive proteins were subjected to the fluorescence recovery after photobleaching (FRAP) assay. Detailed information was provided in Supplementary Table 1. **b** MRNIP-GFP were expressed in HeLa cells. **c** MRNIP-mEGFP protein formed puncta in the nucleus. **d** IF assays showed MRNIP formed puncta in HeLa cells. **e-f** IF assays using 3 individual antibodies were performed to detect MRNIP in HeLa wildtype and MRNIP knock out cells. **g** MRNIP-GFP were not overlapped with nucleolus. HeLa cells were transfected with indicated plasmid and observed with confocal microscopy. For (**b-c, g**), HeLa cells were transfected with indicated plasmid for 24 hours before detection. **h** Co-staining of MRNIP and γ-H2A.X in CRC tissues. MRNIP (Abcam, ab150917) and γ-H2A.X (CST, #80312) antibody were used. White arrow, the merged puncta of MRNIP and γ-H2A.X.

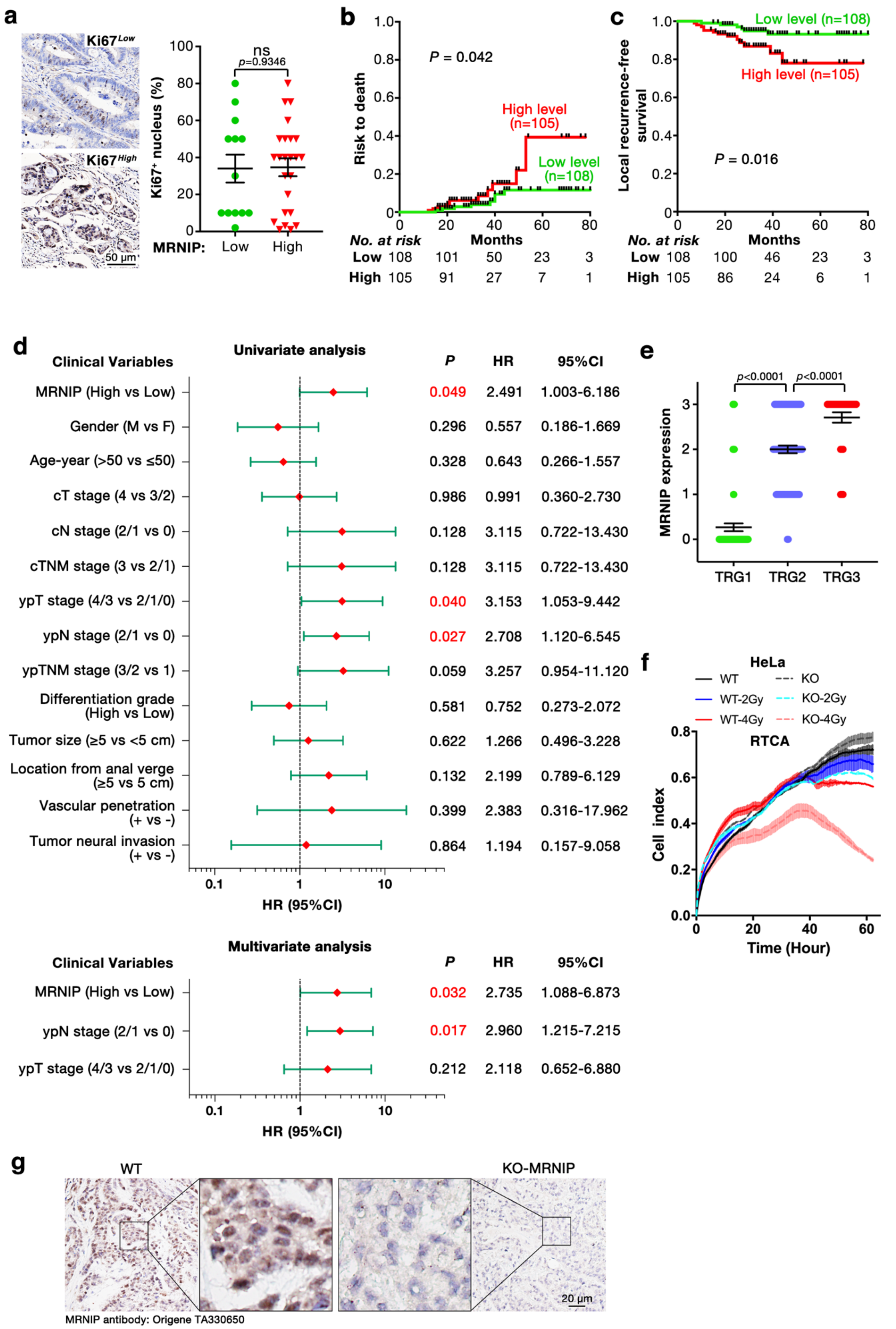

Supplementary Figure 2. High expression of MRNIP was associated with the radioresistance and poor prognosis of CRC patients. a The expression level of MRNIP has

no correlation with the proliferation marker Ki67 level. Data are presented as means ± SEM. MRNIP-Low, *n* = 13; MRNIP-High, *n* = 24. Two-tailed unpaired Student's *t* test. **b-c** Higher MRNIP expression was correlated with shorter survival time of CRC patients. The correlations were analyzed with Kaplan-Meier curve and Log-rank test. **d** The multivariate analysis showed that MRNIP level served as an independent prognostic factor for overall survival of CRC patients. *n* = 213. The hazard ratio (HR) and log-rank *P* value are indicated. MRNIP level, ypN stage and ypT stage were included in the multivariate analysis. **e** Patients with poor response (TRG2-3) to radiotherapy had a higher MRNIP level than patients with a good response (TRG1). Data are presented as means ± SEM. *n* = 82 (TRG1), *n* = 100 (TRG2), *n* = 31 (TRG3). Two-tailed unpaired Student's *t* test. **f** Knocking out of MRNIP has no influence on cell proliferation and sensitized tumor cells to irradiation. Cell viability was examined with RTCA. *n* = 2 biological replicates. **g** IHC assay showed that MRNIP was depleted in the xenografts derived from HeLa-KO-MRNIP cells. ns, no significance.

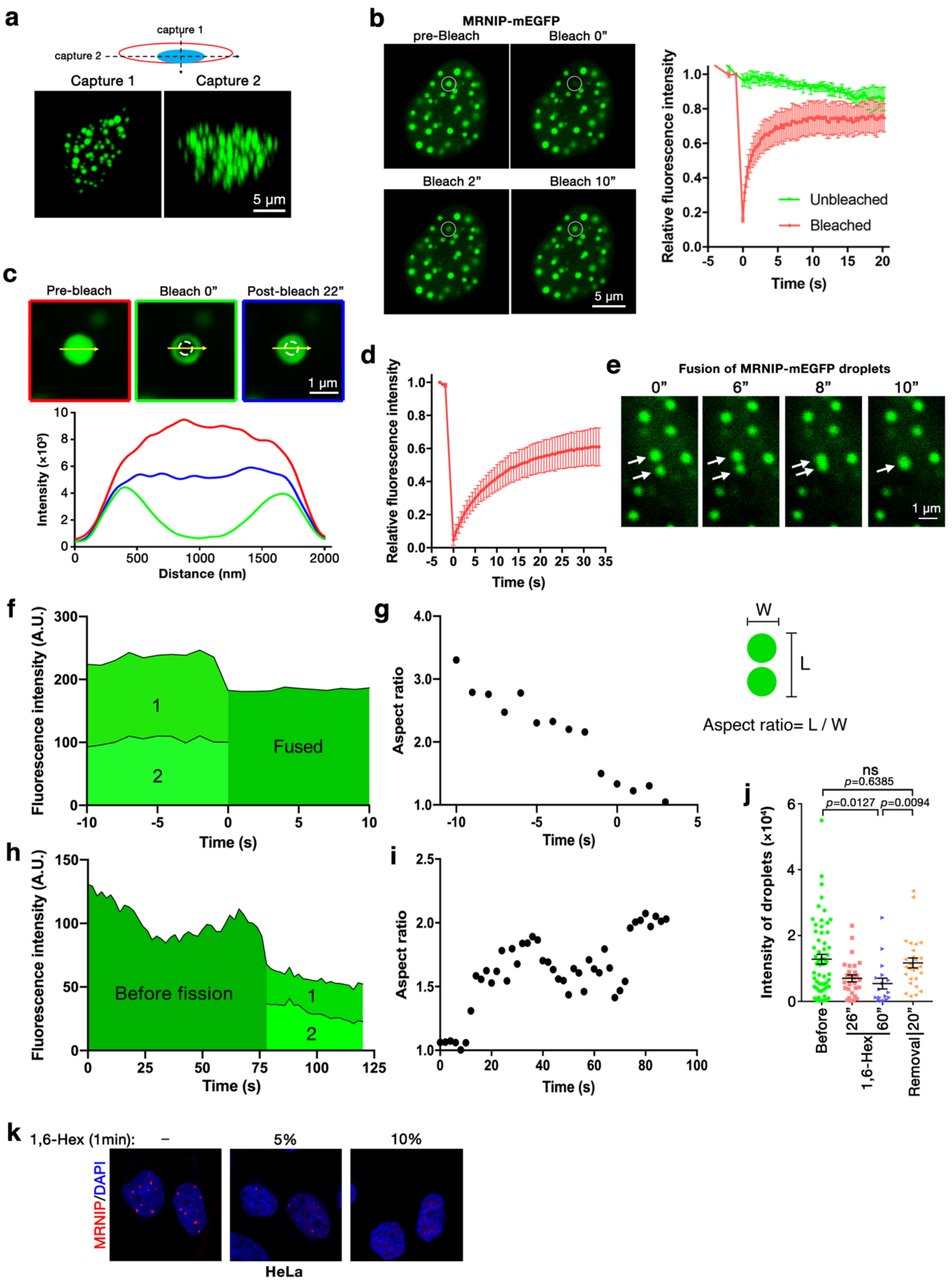

Supplementary Figure 3. MRNIP-GFP formed liquid-like droplets *in vivo*. a 3D capture of MRNIP-GFP in live cells. **b** FRAP of MRNIP-mEGFP in cells. Data are presented as means ± SEM. *n* = 3 foci analysed in 3 independent experiments. **c-d** FRAP of a region within the

droplet in live cell. Data are presented as means ± SEM. *n* = 3 foci analysed in 3 independent experiments. **e** Fusion of different MRNIP-mEGFP droplets was observed in cells. For (**a-e**), HeLa cells were transfected with indicated plasmid for 24 hours before observation. **f-g** the intensity and aspect ratio of droplets in Fig. 2c were calculated. **h-i** the intensity and aspect ratio of droplets in Fig. 2d were calculated. **j** the impact of 1.6-hex on droplets intensity in Fig. 2e. Data are presented as means ± SEM. *n* = 58 (Before), *n* = 28 (1,6-Hex 20”), *n* = 17 (1,6-Hex 60”), *n* = 27 (Removal 20”). Two-tailed unpaired Student’s *t* test. **k** The impact of 1,6-hex on endogenous MRNIP puncta of HeLa cells were examined using IF assay. ns, no significance.

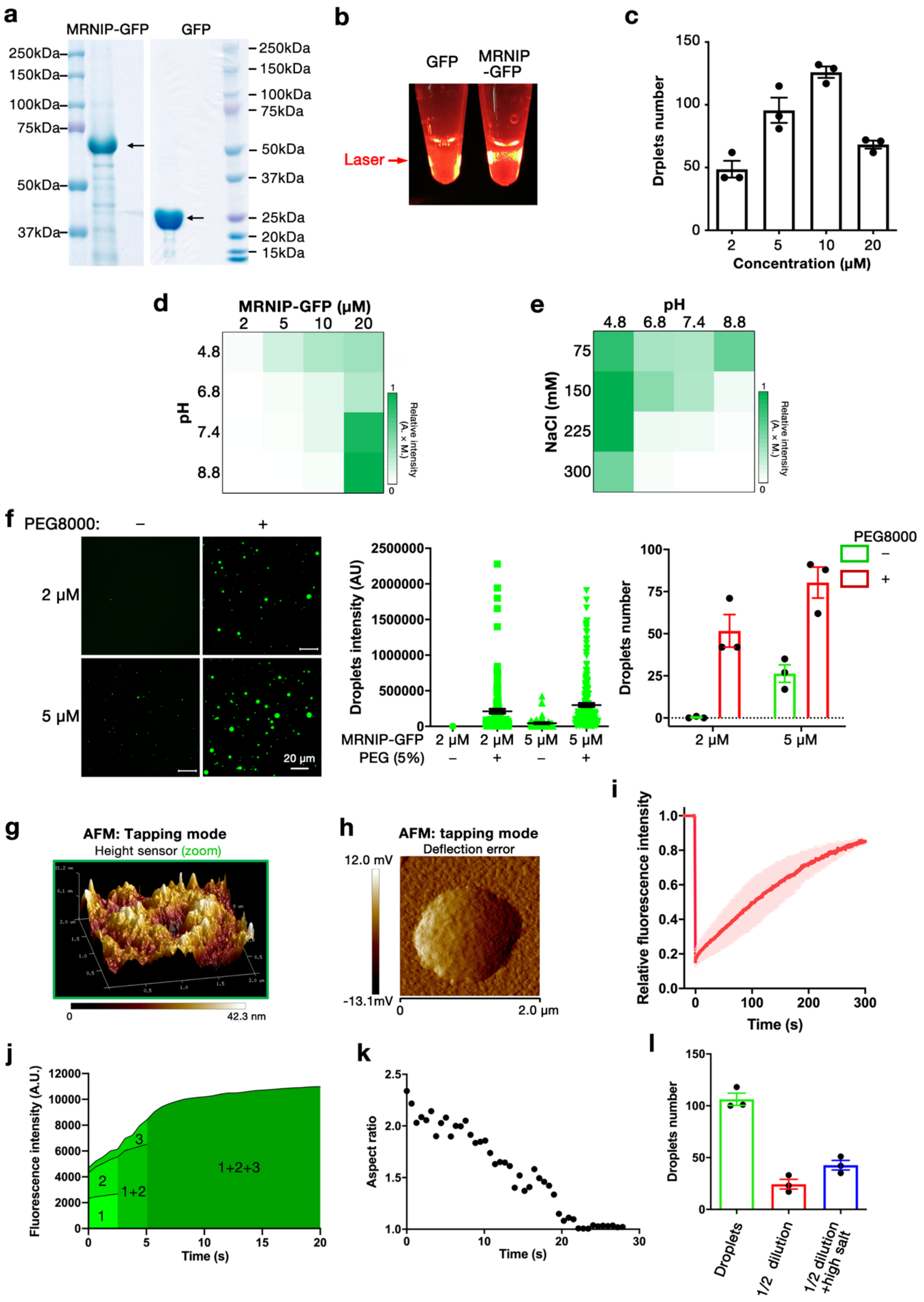

Supplementary Figure 4. Recombinant MRNIP-GFP formed liquid-like droplets *in vitro*.

a Coomassie staining of purified MRNIN-GFP and GFP. **b** Tyndall effect of muddy MRNIP-

GFP solution. Ten micromolar protein was used. **c** The fluorescence intensity of MRNIP droplets in Fig. 2h were calculated. Data are presented as means ± SEM. *n* = 3 biological replicates. **d-e** The impact of protein concentration, NaCl concentration and pH on the formation of MRNIP-GFP droplets. **f** PEG-8000 enhanced the formation of MRNIP-GFP droplets. Data are presented as means ± SEM. *Left panel*: *n* = 1 (2 μ M-PEG), *n* = 155 (2μM+PEG), *n* = 76 (5μM-PEG), *n* = 241 (5μM+PEG); *Right panel*: *n* = 3 biological replicates. **g-h** Characterization of the morphology of MRNIP droplets using AFM in tapping mode. **i** region within the MRNIP-GFP droplets was photobleached, and fluorescence recovered rapidly. Related to Fig. 2l. Data are presented as means ± SEM. *n* = 2 independent experiments. **j-k** The fluorescence intensity and aspect ratio of MRNIP droplets in Fig. 2m were calculated. **l** Droplets number in Fig. 2n were counted. Data are presented as means ± SEM. *n* = 3 biological replicates.

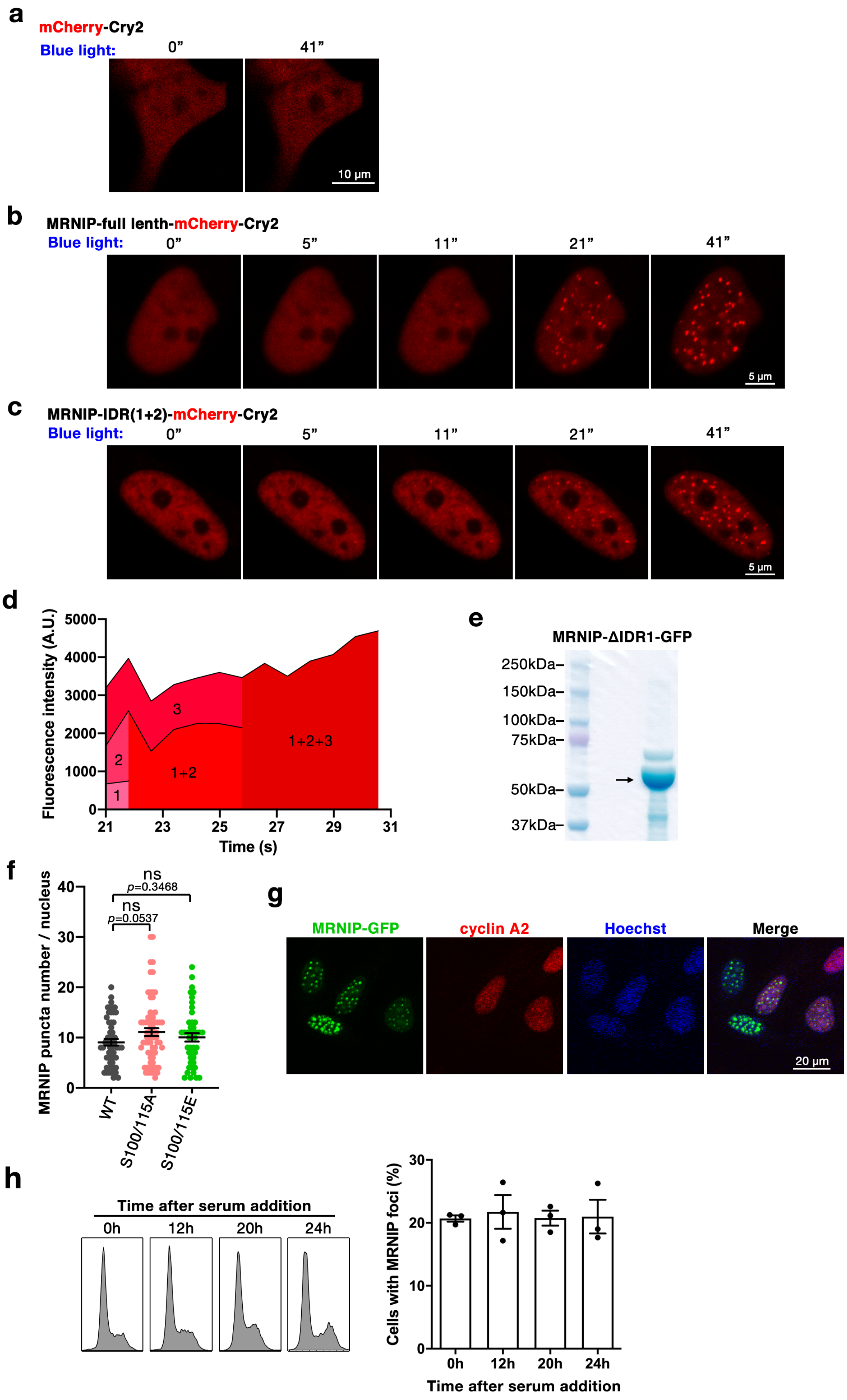

Supplementary Figure 5. The IDR1 is required for LLPS of MRNIP. a Blue light has no

influence on Cry2-mcherry protein. **b-c** MRNIP full length- and IDR(1+2)-Cry2-mCherry protein formed puncta under blue light stimulation (488 nm). **d** The fluorescence intensity of blue light-induced droplets in Fig. 3g were calculated. **e** Coomassie staining of recombinant MRNIP-ΔIDR1-GFP protein. **f** mutating S100/115 to A or E has no impact on MRNIP puncta formation. Data are presented as means ± SEM. *n* = 54 cells (WT), *n* = 69 cells (S100/115A), *n* = 45 cells (S100/115E). Two-tailed unpaired Student's *t* test. **g** MRNIP condensates were presented in both cyclin A2 positive and negative cells. For (**a-d, f-g**), HeLa cells were transfected with indicated plasmid for 24 hours before observation. **h** MRNIP condensates positive cell population remained stable in serum deprivation-releasing model. HeLa cells were cultured in serum free medium for 48 hours before re-adding 15% serum-containing medium. After incubation for indicating time, cells were harvest for FACS or IF assay. Data are presented as means ± SEM. *n* = 3 biological replicates. ns, no significance.

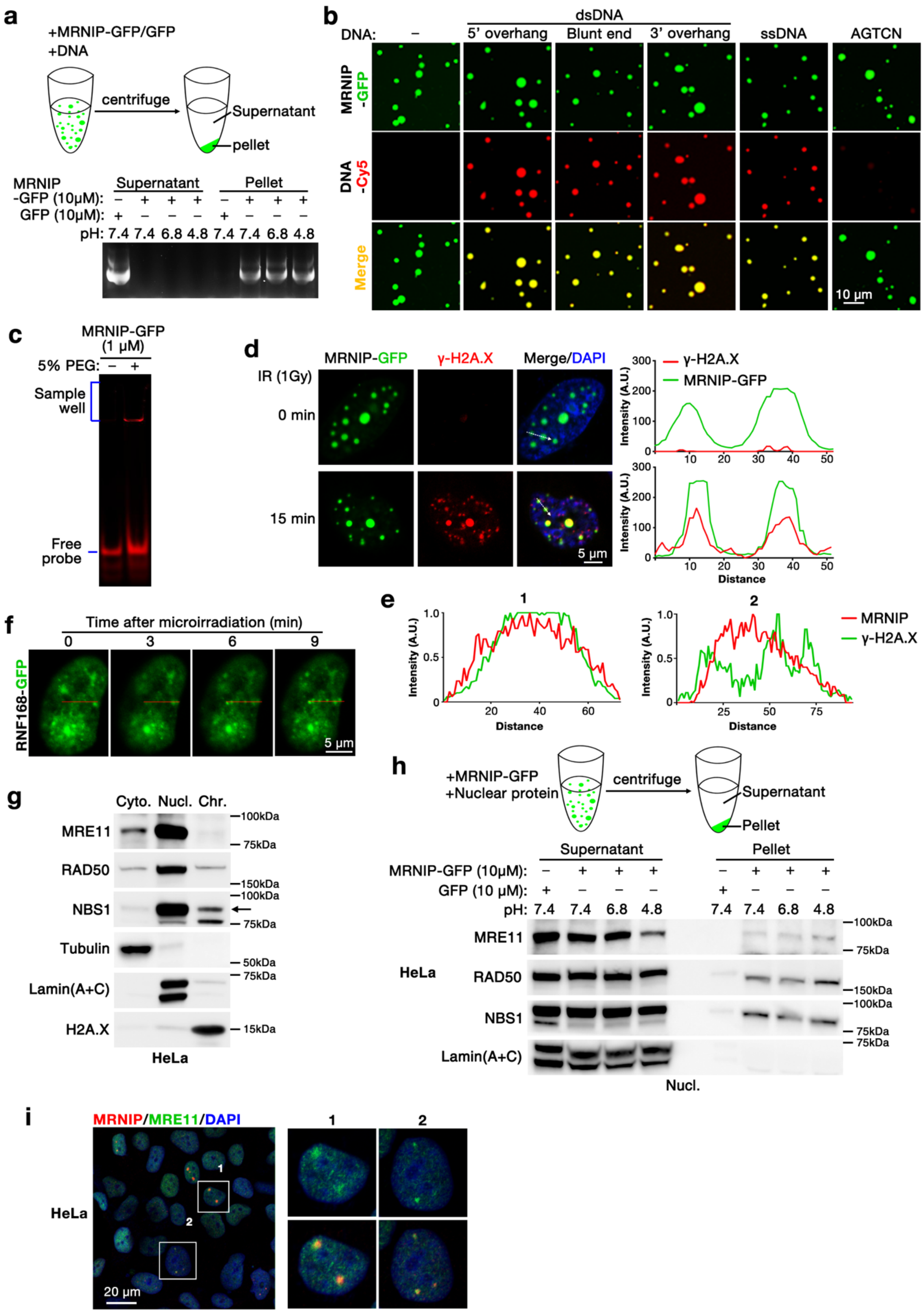

Supplementary Figure 6. MRNIP condensates recruit DNA and the MRN complex. **a** The impact of different pH values on the recruitment of DNA by MRNIP condensates. **b** MRNIP-

GFP incorporated Cy5-tagged DNA in vitro. Ten micromolar MRNIP was incubated with 2 μM Cy5-tagged dsDNA in buffer containing 150 mM NaCl, pH 7.4. **c** MRNIP-GFP droplets recruited and detained DNA in the gel assay. MRNIP (1 μM) was incubated with 0.5 μM Cy5-dsDNA in buffer containing 150 mM NaCl (pH 7.4) with or without 5% PEG8000. **d** γ-H2A.X localized within MRNIP droplets in cells after 15 minutes' recovery after irradiation. HeLa cells stably expressing MRNIP-GFP were irradiated (1Gy) and subjected to γ-H2A.X detection at indicating time. **e** The profile of fluorescence intensity in Fig. 4b was analyzed. **f** microirradiation induced the accumulation of RNF168-GFP at DNA damage site. HeLa cells were transfected with plasmid and incubated with 10 μM BrdU for 24 hours before microirradiation assay. **g** The fractionation of cytoplasmic, nuclear and chromatin protein. α-Tubulin, Lamin A/C and H2A.X were used as the respective internal controls. **h** The impact of different pH values on the recruitment of MRN complexes by MRNIP condensates. **i** IF assay showed that MRNIP puncta was colocalized with MRE11 in HeLa cells. $n = 3$ biological replicates.

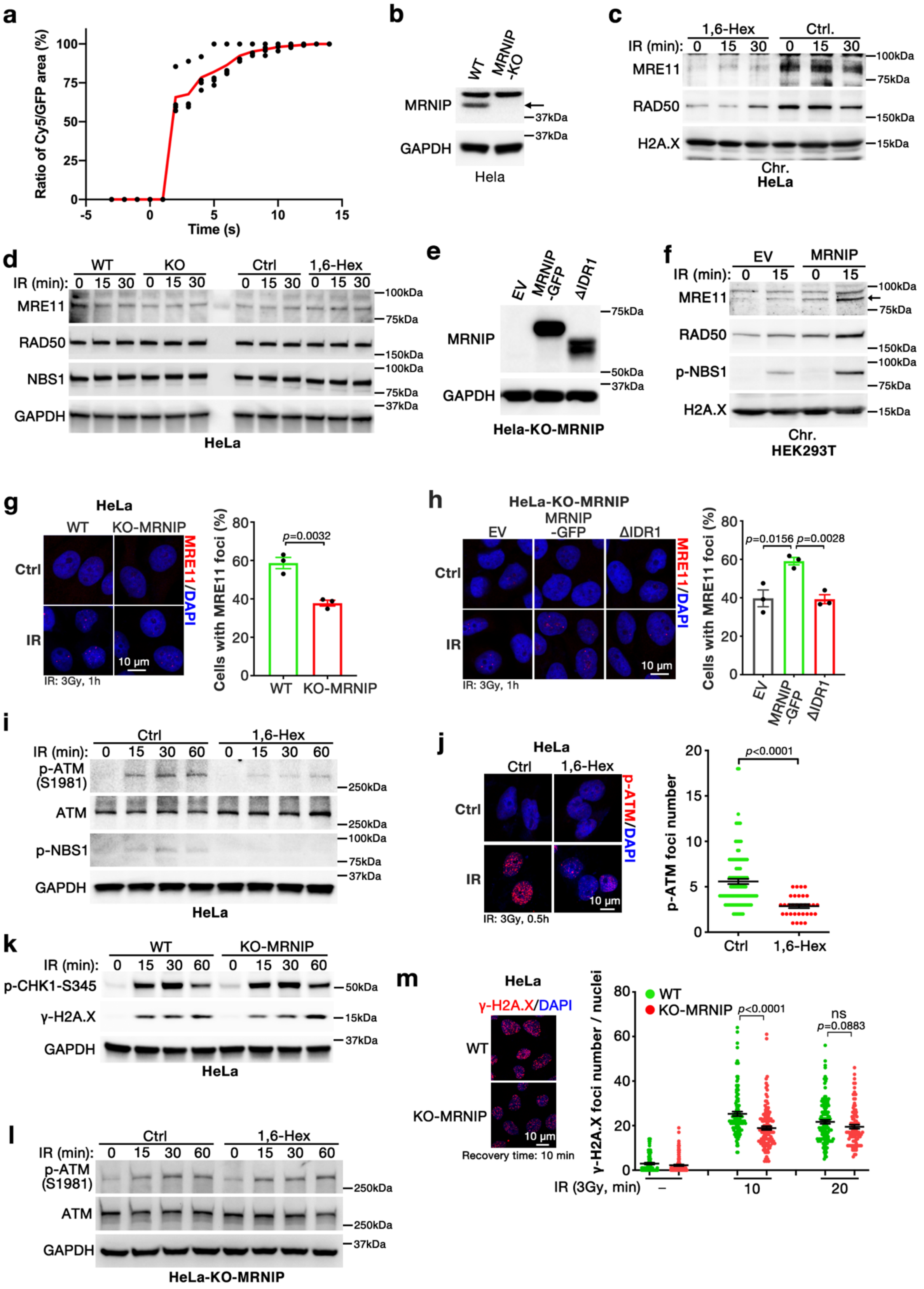

Supplementary Figure 7. MRNIP condensates accelerate the MRN complex loading and ATM activation. a The ratio of Cy5 and GFP-occupied area of each droplet in Fig. 5b was

calculated. **b** MRNIP was knocked out in HeLa cells. **c** 1,6-hexanediol (1.5%) treatment reduced radiation-induced MRN complex binding to chromatin. Chromatin was fractionated at the indicated time after irradiation (10Gy). **d** MRNIP depletion or 1,6-hexanediol (1.5%) treatment had no impact on MRN expression in whole cell lysate. For (**c-d**), 1,6-hexanediol was added to the culture medium just before irradiation. **e** Restoration of MRNIP-GFP or MRNIP-ΔIDR1-GFP in HeLa-KO-MRNIP cells. **f** Overexpression of MRNIP enhanced MRN complex loading after radiation-induced DNA damage. IR: 10 Gy. HEK293T cells were transfected with plasmid for 24 hours before irradiation. **g-h** IF assays were performed to investigate the impact of MRNIP condensates on radiation-induced MRE11 foci. Data are presented as means ± SEM; n = 3 biological replicates. Two-tailed unpaired Student's t test. For (**e, h**), HeLa-KO-MRNIP cells stably expressing sgRNA-resistant MRNIP-GFP, MRNIP-ΔIDR1-GFP (ΔIDR1) and empty vector (EV) were used. **i** The impact of 1,6-hexanediol (1.5%) treatment on radiation-induced ATM and NBS1 phosphorylation. IR: 3Gy. **j** IF assays were performed to analyze the impact of1,6-hexanediol (1.5%) treatment on radiation-induced ATM phosphorylation. HeLa cells were irradiated with 3 Gy. Data are presented as means ± SEM; n = 114 cells (Ctrl), n = 32 cells (1,6-Hex). Two-tailed unpaired Student's t test. **k** The impact of MRNIP depletion on radiation-induced CHK1 phosphorylation. HeLa cells were irradiated with 10 Gy. **l** 1,6-hexanediol had no impact on radiation-induced p-ATM in HeLa-KO-MRNIP cells. IR: 10Gy. **m** MRNIP knockout inhibited the radiation-induced accumulation of γ-H2A.X. Data are presented as means ± SEM. IR^-: n = 105 (WT), n = 113 (KO); IR 10min: n = 115 (WT), n = 130 (KO); IR 20min: n = 125 (WT), n = 96 (KO). Two-tailed unpaired Student's t test. ns, no significance.

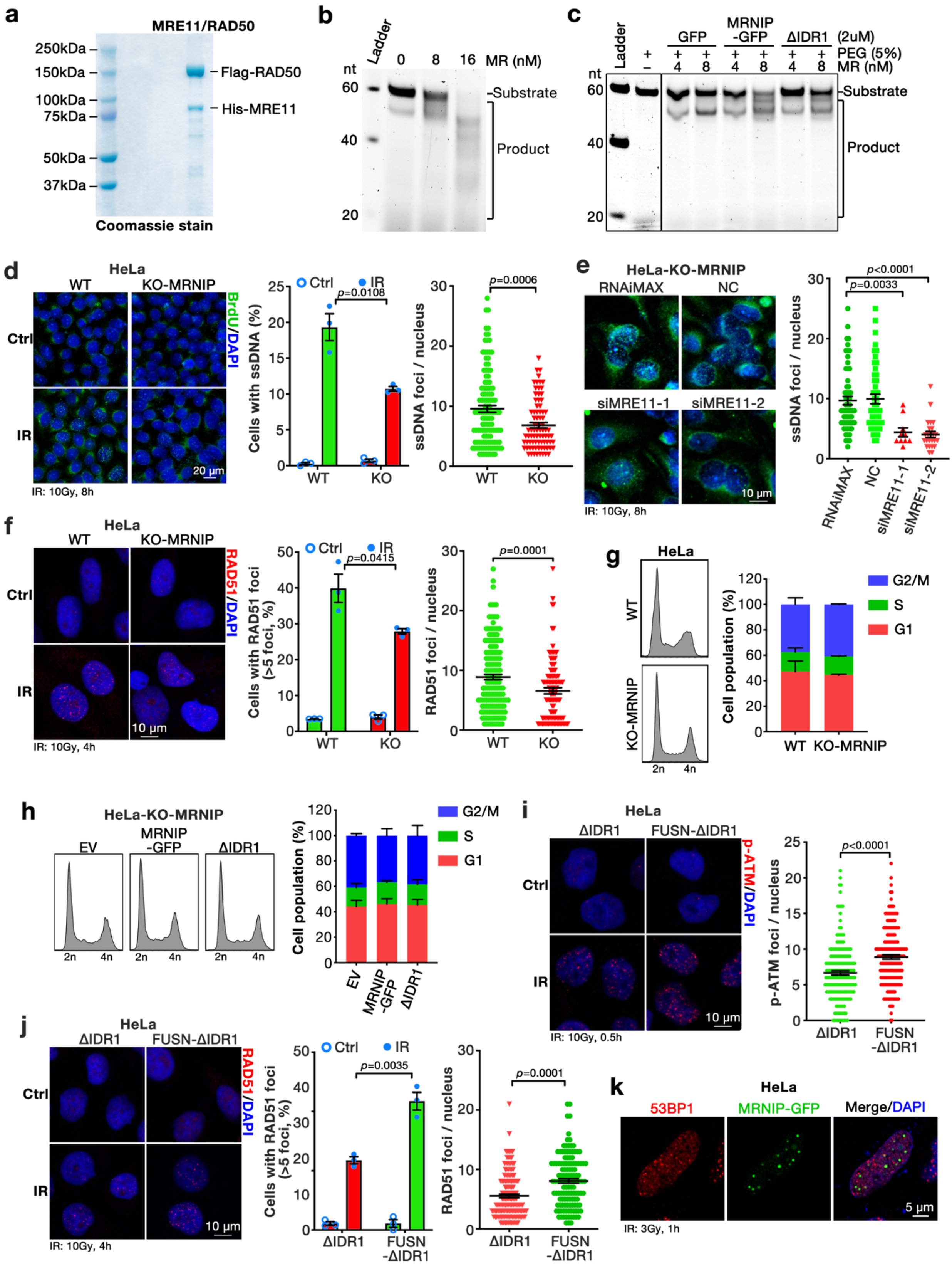

Supplementary Figure 8. MRNIP condensates promote DNA end resection. a Characterization of the MRE11/RAD50 (MR) complex with Coomassie Staining. **b** The

MRE11/RAD50 (MR) complex catalysed the 3′ to 5′ degradation of dsDNA. **c** MRNIP promoted the degradation of dsDNA by the MRE11/RAD50 complex, whereas its IDR1-deleted mutant had no effect in the presence of PEG-8000 (5%). The final concentration of GFP, MRNIP-GFP or ΔIDR1-GFP was 2 μM. **d-e** MRNIP knockout or MRE11 knockdown reduced radiation-induced anti-BrdU foci. Data are presented as means ± SEM. Two-tailed unpaired Student's *t* test. **d,** *Middle panel*: n = 3 biological replicates; *Right panel*: n = 123 (WT), n = 81 (KO-MRNIP). **e,** n = 59 (RNAiMAX), n = 48 (siGFP), n = 10 (siMRE11-1), n = 24 (siMRE11-2). **f** MRNIP knockout reduced radiation-induced anti-RAD51 foci. Data are presented as means ± SEM. Two-tailed unpaired Student's *t* test. *Middle panel*: n = 3 biological replicates; *Right panel*: n = 169 (WT), n = 108 (KO-MRNIP). **g-h** MRNIP condensates had no impact on cell cycle. Data are presented as means ± SEM; n = 2 biological replicates. For (**h**), HeLa cells stably expressing sgRNA-resistant MRNIP-GFP, MRNIP-ΔIDR1-GFP (ΔIDR1) and empty vector (EV) were used. **i-j** FUSN-ΔIDR1 increased p-ATM and RAD51 foci after radiation in HeLa-MRNIP-KO cells. HeLa-KO-MRNIP cells stably expressing sgRNA-resistant MRNIP-ΔIDR1-GFP (ΔIDR1) and FUSN-MRNIP-ΔIDR1-GFP (FUSN-ΔIDR1) were used. Data are presented as means ± SEM; Two-tailed unpaired Student's *t* test. **i**, n = 182 (ΔIDR1), n = 177 (FUSN-ΔIDR1). **j**, *Middle panel*: n = 3 biological replicates. *Right panel*: n = 143 (ΔIDR1), n = 129 (FUSN-ΔIDR1). **k** MRNIP puncta had no association with 53BP1 foci in HeLa cells. HeLa cells stably expressing sgRNA-resistant MRNIP-GFP were irradiated (3Gy) for 1 hour before IF assay.

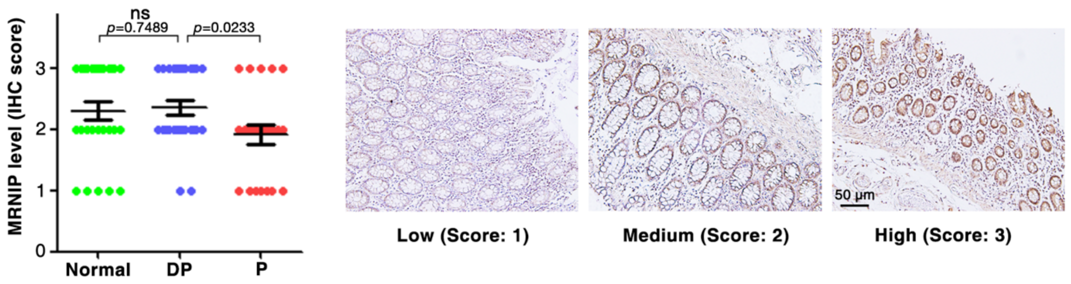

Supplementary Figure 9. MRNIP protein level was reduced in radiation-induced proctitis tissues. *Right panel*, the representative images of IHC score. Normal, adjacent normal colon tissues of CRC patients who didn't receive radiotherapy; P, radiation-induced proctitis tissues; DP, distal proctitis tissues. *Left panel*, Data are presented as means ± SEM; $n = 27$ (Normal), $n = 28$ (DP), $n = 23$ (P). Two-tailed unpaired Student's t test. ns, no significance.

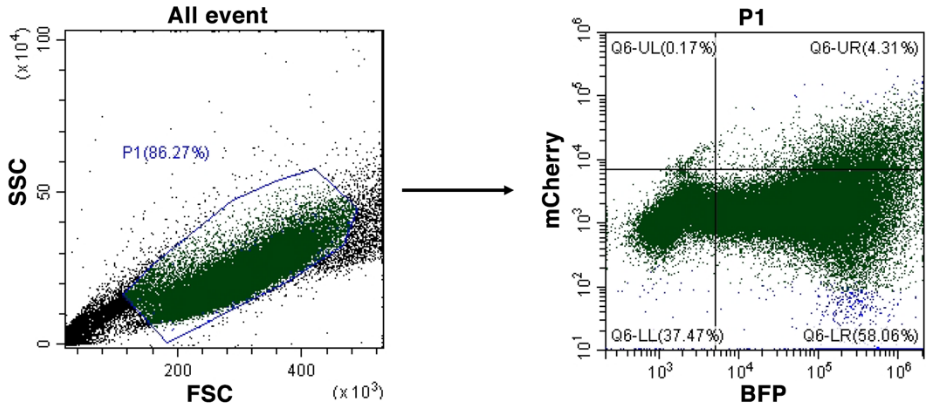

Supplementary Figure 10. Flow cytometry gating. Example of flow cytometry gating strategy used in DSB reporter experiments of Fig. 7a and Fig. 7k.

Supplementary Table 1. Disordered region analysis of DNA repair genes (PONDR)

gene symbol [a]	accession number	PONDR score	puncta?	gene symbol	accession number	PONDR score	puncta?	gene symbol	accession number	PONDR score	puncta?
HMGA2	NP_003474.1	0.998	Yes [b]	HMGA1	NP_665908.1	0.9975	Yes	HMGN1	NP_004956.5	0.9945	No
TNP1	NP_003275.1	0.9935	Yes (nucleoli[c])	NUCKS1	NP_073568.2	0.9879	No	PAGR1	NP_078792.1	0.9287	No
HMGB2	NP_002120.1	0.9214	No [b]	NCOA6	NP_054790.2	0.8955	No	INO80B	NP_112578.2	0.8924	Yes (nucleoli)
FUS	NP_004951.1	0.8894	No	GGN	NP_689870.3	0.8784	Yes	INO80E	NP_775889.1	0.8578	Yes (nucleoli)
RAD51AP1	NP_006470.1	0.8569	Yes	HMGB1	NP_002119.1	0.8511	No	TNKS1BP1	NP_203754.2	0.8511	No
PCLAF	NP_055551.1	0.8257	No	CEP164	NP_055771.4	0.8247	No	BOD1L1	NP_683692.2	0.8247	No
SFPQ	NP_005057.1	0.8238	No	RNF169	NP_001092108.1	0.8226	No	CLSPN	NP_071394.2	0.8164	No
NOP53	NP_056525.2	0.8076	Yes (nucleoli)	RNF111	NP_060080.6	0.8075	No	MDC1	NP_055456.2	0.8053	No
FMN2	NP_064450.3	0.7987	No	CDCA5	NP_542399.1	0.7976	No	CEBPG	NP_001797.1	0.7911	No
SLX4	NP_115820.2	0.788	No	RBM14	NP_006319.1	0.7845	Yes	SYCP1	NP_003167.2	0.7808	No
DEK	NP_003463.1	0.7803	No	RNF168	NP_689830.2	0.7764	Yes	EMSY	NP_001287872.1	0.7752	No
TFPT	NP_037474.1	0.7721	No	TP53BP1	NP_001135452.1	0.7689	Yes	SFR1	NP_660290.3	0.7688	No
PPP4R2	NP_777567.1	0.7681	No	SETD2	NP_054878.5	0.7616	No	RTEL1-TNFRSF6B	PNJ02870.1	0.7589	No
UIMC1	NP_001186227.1	0.7576	Yes	CETN2	NP_004335.1	0.7517	No	RHNO1	NP_001239428.1	0.7485	No
POLD3	NP_006582.1	0.7477	Yes	EP300	NP_001420.2	0.7475	No	MRNIP	NP_057259.2	0.7469	Yes
PTTG1	NP_004210.1	0.7369	Yes	FOXM1	NP_068772.2	0.7344	No	NONO	NP_031389.3	0.7302	No
RBBP8	NP_002885.1	0.7281	Yes	CYREN	NP_001350258.1	0.726	No	TWIST1	NP_000465.1	0.7195	No
FAAP20	NP_872339.2	0.7185	No	TICRR	NP_689472.3	0.7129	No	ATRX	NP_000480.3	0.7081	No
SWI5	NP_001305018.1	0.706	No	CETN1	NP_004057.1	0.706	No	CHAF1A	NP_005474.2	0.6967	
INO80D	NP_060229.3	0.6925		ETAA1	NP_061875.2	0.6916		BRCA1	NP_009225.1	0.6894	
REXO4	NP_065118.2	0.6893		RAD18	NP_064550.3	0.6886		CCDC155	NP_653289.3	0.6861	
CBX8	NP_065700.1	0.686		TP53	NP_000537.3	0.6849		ERCC5	NP_000114.2	0.6847	
RAD23B	NP_002865.1	0.6839		FIGNL2	NP_001013712.4	0.6826		REC8	NP_001041670.1	0.6813	
NFRKB	NP_001137307.1	0.6789		JMY	NP_689618.4	0.6761		CDC5L	NP_001244.1	0.6691	
AXIN2	NP_004646.3	0.6684		PAXX	NP_899064.1	0.6679		HSF1	NP_005517.1	0.6657	
ISY1	NP_065752.1	0.6642		NPM1	NP_002511.1	0.6614		EPC2	NP_056445.3	0.6601	
TAOK3	NP_057365.3	0.66		SMC1A	NP_006297.2	0.6594		TERF2	NP_005643.2	0.6591	
FAM168A	NP_055974.1	0.6589		SLF2	NP_060591.3	0.6569		SEM1	NP_001188379.1	0.6565	
RBM17	NP_116294.1	0.6554		APLF	NP_775816.1	0.6545		NPAS2	NP_002509.2	0.6544	
SIX6OS1	NP_777638.3	0.6517		SPIRE1	NP_064533.3	0.6495		CHCHD4	NP_001091972.1	0.6487	
RFC1	NP_002904.3	0.6486		RAD50	NP_005723.2	0.6486		TCEA1	NP_006747.1	0.6484	
BACH1	NP_001177.1	0.6475		RAD23A	NP_005044.1	0.6469		WAS	NP_000368.1	0.6458	
AUNIP	NP_076942.1	0.6455		NSMCE2	NP_775956.1	0.6417		ZNF830	NP_443089.3	0.6411	
RAD21	NP_006256.1	0.6363		BIVM-ERCC5	NP_001191354.1	0.6335		PWWP3A	NP_116242.3	0.6303	
TERF2IP	NP_061848.2	0.627		SMC3	NP_005436.1	0.6242		TRIM28	NP_005753.1	0.6234	
ABL1	NP_005148.2	0.6216		EYA1	NP_000494.2	0.6182		NABP2	NP_076973.1	0.6176	
DMAP1	NP_061973.1	0.6173		XRCC1	NP_006288.2	0.6168		RAD52	NP_602296.2	0.6162	
RMI2	NP_689521.1	0.6135		ALKBH5	NP_060228.3	0.6131		INO80C	NP_919257.2	0.6129	
RIF1	NP_060621.3	0.6102		E9PQ18	CCP83115	0.6094		INIP	NP_067041.1	0.6085	
MBD4	NP_001263199.1	0.6079		EYA4	NP_004091.3	0.6071		POLR2K	NP_005025.1	0.6047	
DTL	NP_057532.4	0.6042		FANCE	NP_068741.1	0.6014		RNF113A	NP_008909.1	0.6005	
TENT4A	NP_008930.2	0.6		NIPBL	NP_597677.2	0.5982		EME1	NP_689676.2	0.5958	
SHLD1	NP_689717.2	0.5957		WDR33	NP_060853.3	0.5953		SPIRE2	NP_115827.1	0.5948	
EXO1	NP_569082.2	0.5929		NBN	NP_002476.2	0.5927		UVSSA	NP_065945.2	0.5924	
ESCO2	NP_001017420.1	0.592		ATXN3	NP_004984.2	0.5896		PMS2P11	Q13670.1	0.5895	
A0A1W2PQ90	Q13670.1	0.5895		POLR2D	NP_004796.1	0.5879		CGAS	NP_612450.2	0.5862	
SIRT1	NP_036370.2	0.5846		TP73	NP_005418.1	0.5827		CENPS	NP_954988.1	0.5821	
APBB1	NP_001155.1	0.5807		MORF4L2	NP_036418.1	0.5804		RNF8	NP_003949.1	0.5801	

SPRTN	NP_114407.3	0.578		ERCC6L2	NP_064592.2	0.577		ATRIP	NP_569055.1	0.5766	
NSD2	NP_001035889.1	0.5761		REV3L	NP_002903.3	0.576		NEIL1	NP_078884.2	0.5739	
SUMO1	NP_003343.1	0.5739		ANKLE1	NP_689576.6	0.5738		CSB-PGBD3	NP_000115.1	0.5738	
ERCC6	NP_000115.1	0.5738		FIGN	NP_060556.2	0.5732		KIN	NP_036443.1	0.571	
BLM	NP_000048.1	0.5696		TEX12	NP_112565.1	0.569		SPATA22	NP_001164169.1	0.5689	
BARD1	NP_000456.2	0.5687		RECQL4	NP_004251.3	0.5684		PARP10	NP_116178.2	0.5682	
POLD4	NP_066996.3	0.5681		MCM9	NP_060166.2	0.5679		BRCA2	NP_000050.2	0.5669	
LIG1	NP_000225.1	0.5669		SMC6	NP_001135758.1	0.5663		PALB2	NP_078951.2	0.5651	
PARG	NP_003622.2	0.5651		EYA3	NP_001981.2	0.5636		RECQL5	NP_004250.4	0.5625	
PML	NP_150241.2	0.558		RPAIN	NP_001028174.2	0.5575		RFWD3	NP_060594.3	0.5571	
SSRP1	NP_003137.1	0.557		INO80	NP_060023.1	0.5555		TONSL	NP_038460.4	0.555	
HUWE1	NP_113584.3	0.5546		KIF22	NP_015556.1	0.5518		UBR5	NP_056986.2	0.5493	
SWSAP1	NP_787067.2	0.5481		FANCM	NP_065988.1	0.5475		POLH	NP_006493.1	0.5471	
HINFP	NP_945322.1	0.547		PMS2P1	A4D2B8	0.5466		MNAT1	NP_002422.1	0.5464	
XPC	NP_004619.3	0.546		TDG	NP_003202.3	0.5458		MEIOC	NP_001138552.2	0.5453	
POLR2F	NP_068809.1	0.5447		POLK	NP_057302.1	0.5441		DCLRE1A	NP_055696.3	0.5427	
SETX	NP_055861.3	0.5392		TEX15	NP_001337091.1	0.5388		MRE11	NP_005582.1	0.5384	
USP10	NP_005144.2	0.5381		XRCC4	NP_071801.1	0.5374		RPS27A	NP_001170884.1	0.5342	
GEN1	NP_001123481.3	0.5339		TRIP12	NP_001335252.1	0.5334		MPG	NP_001015052.1	0.5332	
EME2	NP_001244299.1	0.5328		GINS4	NP_115712.1	0.5328		DTX3L	NP_612144.1	0.5309	
NEIL2	NP_659480.1	0.5304		SMC5	NP_055925.2	0.5303		POLI	NP_009126.2	0.5295	
SMARCAD1	NP_064544.2	0.5283		SLX1A	NP_001014999.1	0.5282		NABP1	NP_001026886.1	0.5282	
NSMCE4A	NP_060085.2	0.528		PAXIP1	NP_031375.3	0.5276		BABAM1	NP_054892.2	0.5276	
REV1	NP_057400.1	0.5271		POLR2A	NP_000928.1	0.5233		FEN1	NP_004102.1	0.5225	
PMS2P5	A8MQ11	0.5207		ABRAXAS1	NP_620775.2	0.52		NSMCE3	NP_619649.1	0.5189	
STUB1	NP_005852.2	0.5161		SMARCAL1	NP_054859.2	0.5159		MLH3	NP_001035197.1	0.5148	
MAGEF1	NP_071432.2	0.5142		UHRF1	NP_001041666.1	0.5121		MCRS1	NP_006328.2	0.512	
RAD21L1	NP_001130038.2	0.5119		KAT5	NP_874369.1	0.5111		UBE2B	NP_003328.1	0.5082	
XPA	NP_000371.1	0.5076		HIST3H3	NP_003484.1	0.5076		MUS81	NP_079404.3	0.5075	
H2AFX	NP_002096.1	0.5073		SIRT6	NP_057623.2	0.5063		IGHMBP2	NP_002171.2	0.5055	
EYA2	NP_005235.3	0.5052		TRIM25	NP_005073.2	0.5047		MTA1	NP_004680.2	0.5042	
RMI1	NP_001345220.1	0.5039		TOPBP1	NP_008958.2	0.5031		KDM2A	NP_036440.1	0.5024	
MARF1	NP_055462.2	0.5019		BCCIP	NP_510868.1	0.5011		PARP1	NP_001609.2	0.501	
CIB1	NP_006375.2	0.5008		SPIDR	NP_001073863.1	0.5003		DCLRE1C	NP_001029027.1	0.4998	
KDM4D	NP_060509.2	0.4995		UBE2A	NP_003327.2	0.4985		FZR1	NP_057347.2	0.4949	
PIF1	NP_001273425.1	0.4935		ZNF365	NP_055766.2	0.4934		RTEL1	NP_057518.1	0.493	
MUTYH	NP_001121897.1	0.4929		CHAF1B	NP_005432.1	0.4926		CINP	NP_116019.1	0.4922	
FIGNL1	NP_001333494.1	0.4917		PMS2	NP_000526.2	0.4916		PARP3	NP_005476.4	0.4911	
KDM1A	NP_055828.2	0.4892		TIMELESS	NP_003911.2	0.4885		TTC5	NP_612385.2	0.4884	
ASCC2	NP_115580.2	0.4876		POLM	NP_037416.1	0.4867		UBE2T	NP_054895.1	0.486	
UBA52	NP_001029102.1	0.4856		NTHL1	NP_002519.2	0.4855		PDS5B	NP_055847.1	0.4827	
PARP2	NP_001036083.1	0.4801		RNF138	NP_057355.2	0.479		BRIP1	NP_114432.2	0.4775	
RAD9A	NP_004575.1	0.4771		TDP1	NP_060789.2	0.477		PPIE	NP_001181936.1	0.4767	
CDC7	NP_003494.1	0.4765		SUPT16H	NP_009123.1	0.4764		ERCC1	NP_001974.1	0.4752	
UNG	NP_550433.1	0.4745		YY1	NP_003394.1	0.4733		ZBTB7A	NP_056982.1	0.4702	
HIST3H2A	NP_254280.1	0.4688		SHLD3	NP_001352270.1	0.4683		ALKBH2	NP_001138846.1	0.4683	
HIST1H4	NP_003529.1	0.4667		UBE2V2	NP_003341.1	0.4666		ACTR5	NP_079131.3	0.4657	
TREX1	NP_338599.1	0.4646		CUL4B	NP_001073341.1	0.4643		PMS1	NP_000525.1	0.4642	
RAD17	NP_579916.1	0.4629		CSNK1E	NP_689407.1	0.4629		CHEK2	NP_009125.1	0.4628	
TFIP11	NP_036275.1	0.462		APEX2	NP_055296.2	0.4612		RPA2	CAG29344	0.4587	
MSH6	NP_000170.1	0.4573		APTX	NP_001182177.1	0.4568		MEN1	NP_570711.1	0.4567	
POLQ	NP_955452.3	0.456		MORF4L1	NP_006782.1	0.4553		FAN1	NP_055782.3	0.4549	
PIAS4	NP_056981.2	0.4539		POLL	NP_001167555.1	0.4533		UFD1	NP_005650.2	0.4533	
GPS1	NP_001308018.1	0.4531		COPS7A	NP_001157566.1	0.453		FAAP100	NP_079437.5	0.4522	
EEPD1	NP_085139.2	0.4521		NEIL3	NP_060718.3	0.4518		POLR2C	NP_116558.1	0.4517	

DDX11	NP_085911.2	0.4513		MGME1	NP_443097.1	0.4507		EXD2	NP_001180289.1	0.4484	
ASF1A	NP_054753.1	0.447		UBE2W	NP_060769.5	0.4455		LIG3	NP_039269.2	0.4442	
GTF2H1	NP_005307.1	0.44		MGMT	NP_002403.3	0.4397		PARP4	NP_006428.2	0.4396	
USP43	NP_694942.3	0.4389		APEX1	NP_001632.2	0.4383		WRNIP1	NP_064520.2	0.4376	
CHD1L	NP_004275.4	0.4374		NHEJ1	NP_079058.1	0.4372		AP5S1	NP_060817.1	0.4365	
DCLRE1B	NP_073747.1	0.4356		MLH1	NP_000240.1	0.4351		HELB	NP_387467.2	0.4349	
POLA1	NP_001317289.1	0.4344		PMS2P2	O95744	0.4342		ALKBH3	NP_631917.1	0.4336	
UBE2V1	NP_001027459.1	0.4335		ENDOV	NP_775898.2	0.4331		CDC14B	NP_201588.1	0.4325	
EID3	NP_001008395.1	0.4325		FGF10	NP_004456.1	0.4313		POLN	NP_861524.2	0.4312	
USP1	NP_003359.3	0.43		CHRNA4	NP_000735.1	0.4298		MSH3	NP_002430.3	0.4297	
INTS3	NP_075391.3	0.4296		VCP	NP_009057.1	0.429		PNKP	NP_009185.2	0.4279	
HDAC10	NP_114408.3	0.4272		COPS7B	NP_073567.1	0.4266		PARPBP	NP_060385.3	0.4243	
POLB	NP_002681.1	0.4235		TDP2	NP_057698.2	0.4231		SHPRH	NP_001036148.2	0.4225	
ERCC3	NP_000113.1	0.4222		POLG	NP_002684.1	0.4206		COPS2	NP_004227.1	0.4205	
ADPRHL2	NP_060295.1	0.419		MCM8	NP_115874.3	0.4189		ZRANB3	NP_115519.2	0.4175	
RAD54L	NP_003570.2	0.4163		ISG15	NP_005092.1	0.4158		UBB	NP_061828.1	0.4152	
OTUB1	NP_060140.2	0.4151		PRIMPOL	NP_689896.1	0.4146		PDS5A	NP_001093869.1	0.4143	
ERCC4	NP_005227.1	0.4132		USP28	NP_001333181.1	0.4127		ACTR8	NP_075050.3	0.4123	
SMUG1	NP_001338175.1	0.4122		RFC4	NP_853551.1	0.412		EGFR	NP_005219.2	0.4119	
DHX9	NP_001348.2	0.4109		TIGAR	NP_065108.1	0.4106		RAD9B	NP_001273464.1	0.4099	
FANCG	NP_004620.1	0.4095		WRAP53	NP_060551.2	0.4095		SETMAR	NP_006506.3	0.409	
ATP23	NP_150592.1	0.4046		CCNH	CAG28586	0.404		RUVBL2	NP_006657.1	0.4038	
GINS2	NP_057179.1	0.4038		BRCC3	NP_001018065.1	0.4031		WDR70	NP_060504.1	0.4019	
UBC	NP_066289.3	0.4008		HELQ	NP_598375.2	0.3982		TREX2	NP_542432.2	0.3966	
RADX	NP_060485.4	0.3966		ASCC1	NP_001185729.1	0.3965		UBE2L6	NP_004214.1	0.3961	
ALKBH1	NP_006011.2	0.3959		UBE2U	NP_001353161.1	0.3956		HUS1B	NP_683762.2	0.3948	
RPS3	NP_000996.2	0.3944		RAD54B	NP_036547.1	0.3943		CENPX	NP_001257935.1	0.3941	
SHLD2	NP_001317041.1	0.394		PRPF19	NP_055317.1	0.3939		RPA4	NP_037479.1	0.3939	
MSH4	NP_002431.2	0.3939		RUVBL1	NP_003698.1	0.3906		CDKN2D	NP_001791.1	0.3901	
SLC30A9	NP_006336.3	0.3899		RPA1	NP_002936.1	0.3891		UCHL5	NP_001186190.1	0.389	
CHEK1	NP_001107594.1	0.3887		HERC2	NP_004658.3	0.3881		PRKCG	NP_002730.1	0.3878	
POLG2	NP_009146.2	0.3875		SLF1	NP_115666.2	0.3871		RNASEH2A	NP_006388.2	0.3869	
PRMT6	NP_060607.2	0.3866		FANCF	NP_073562.1	0.386		RECQL	NP_002898.2	0.3846	
PPP5C	NP_006238.1	0.3836		AP5Z1	NP_055670.1	0.3826		DDB2	NP_000098.1	0.382	
SMCHD1	NP_056110.2	0.3809		OGG1	NP_002533.1	0.3793		CDC45	NP_003495.1	0.3787	
PSMD14	NP_005796.1	0.378		PARK7	NP_009193.2	0.3775		FAAP24	NP_689479.1	0.3768	
ERCC8	NP_000073.1	0.3742		FANCI	NP_001106849.1	0.3733		NUDT16	NP_689608.2	0.3726	
CUL4A	NP_001008895.1	0.3724		POLD1	NP_001243778.1	0.3716		FTO	NP_001073901.1	0.3711	
COPS5	NP_006828.2	0.3708		CRY2	NP_066940.3	0.3704		GTF2H2C	NP_001092198.1	0.3686	
SMG1	NP_055907.3	0.3686		FANCD2	NP_001018125.1	0.3676		DNTT	NP_004079.3	0.3657	
EXO5	NP_001333882.1	0.3644		DMC1	NP_008999.2	0.3633		POLR2I	NP_006224.1	0.36	
POLD2	NP_006221.2	0.3599		FANCA	NP_000126.2	0.3599		GTF2H2	NP_001506.1	0.3594	
RAD51	NP_002866.2	0.3587		USP47	NP_001269588.1	0.3578		RAD51D	NP_002869.3	0.357	
NSMCE1	NP_659547.2	0.3564		ZSWIM7	NP_001036162.1	0.3561		UBE2NL	NP_001013007.1	0.3557	
CDK9	NP_001252.1	0.3556		POLR2H	NP_006223.2	0.3552		GADD45A	NP_001915.1	0.3542	
UVRAG	NP_003360.2	0.353		RAD51C	NP_478123.1	0.3522		UBE2D3	NP_871620.1	0.3522	
FBH1	NP_835363.1	0.3512		POLR2E	NP_002686.2	0.3511		ZBTB1	NP_001116801.1	0.3509	
RCHY1	NP_056251.2	0.3502		MSH2	NP_000242.1	0.3501		BTG2	NP_006754.1	0.3499	
POLE2	NP_002683.2	0.3494		PCNA	NP_872590.1	0.3476		PARP9	NP_001139576.1	0.347	
PRKDC	NP_008835.5	0.347		NPLOC4	NP_060391.2	0.3469		POLE	NP_006222.2	0.3462	
DDX1	NP_004930.1	0.3456		AQR	NP_055506.1	0.3448		ZFYVE26	NP_056161.2	0.3433	
POLR2J	NP_006225.1	0.3429		SAMHD1	NP_056289.2	0.3416		UPF1	NP_002902.2	0.341	
LIG4	NP_002303.2	0.3399		NUDT16L1	NP_115725.1	0.3396		USP51	NP_958443.1	0.339	

COPS3	NP_003644.2	0.3376		UBE2N	NP_003339.1	0.3368		SPO11	NP_036576.1	0.3358	
XRCC6	NP_001460.1	0.3355		RFC3	NP_002906.1	0.3352		RAD51B	NP_001308743.1	0.3346	
CDK7	NP_001790.1	0.3331		MSH5	NP_751898.1	0.3325		POLR2B	NP_000929.1	0.3324	
ACTL6A	NP_004292.1	0.3324		ATM	NP_000042.3	0.3288		MEIOB	NP_001157032.1	0.328	
NUDT1	NP_945188.1	0.3259		HUS1	NP_004498.1	0.3248		CDK1	NP_001777.1	0.3247	
USP45	NP_001332951.1	0.322		COPS6	NP_006824.2	0.3213		ASTE1	NP_054784.2	0.3168	
COPS4	NP_057213.2	0.3167		TRRAP	NP_003487.1	0.3163		DNA2	NP_001073918.2	0.315	
FANCB	NP_001018123.1	0.3132		MMS19	NP_071757.4	0.3131		GTF2H3	NP_001507.2	0.3123	
ASCC3	NP_006819.2	0.311		RBX1	NP_055063.1	0.3108		ATR	NP_001175.2	0.3097	
DDB1	NP_001914.3	0.3094		XAB2	NP_064581.2	0.3094		WRN	NP_000544.2	0.3064	
BABAM2	NP_954661.1	0.3061		WDHD1	NP_009017.1	0.3048		PSME4	NP_055429.2	0.3043	
RPA3	NP_002938.1	0.3035		RRM2B	NP_056528.2	0.2993		RFC2	NP_852136.1	0.2992	
RAD1	NP_002844.1	0.2976		COPS8	NP_006701.1	0.296		GTF2H4	NP_001508.1	0.2959	
TMEM189-UBE2V1	NP_954673.1	0.2947		TRIP13	NP_004228.1	0.2947		XRCC3	NP_005423.1	0.2945	
RFC5	NP_031396.1	0.2918		POLR2G	NP_002687.1	0.2868		KLHL15	NP_085127.2	0.2867	
ACTR2	NP_005713.1	0.2848		WDR48	NP_065890.1	0.2848		ERCC2	NP_000391.1	0.2839	
FBXO6	NP_060908.1	0.2823		CDK2	NP_001789.2	0.281		MAD2L2	NP_006332.3	0.2798	
XRCC2	NP_005422.1	0.275		MMS22L	NP_001337528.1	0.2748		PPP4C	NP_002711.1	0.274	
FANCC	NP_000127.2	0.2696		FANCL	NP_060532.2	0.2677		TMEM161A	NP_060284.1	0.2643	
GTF2H5	NP_997001.1	0.2605		USP7	NP_003461.2	0.25		MCMDC2	NP_775789.3	0.2476	
XRCC5	NP_066964.1	0.2265		POLR2L	NP_066951.1	0.2128		USP3	NP_006528.2	0.2001	
MC1R	NP_002377.4	0.1767		ZMPSTE24	NP_005848.2	0.1136					

[a] Fifty-six high-scoring (>0.7) genes were highlighted with green.

[b] Yes, puncta forming; No, no puncta forming.

[c] Nucleoli, localizing to nucleoli.
