# Supplementary material for: MRNIP condensates promote DNA double-strand break sensing and end resection
Source: Nat Commun. 2022 May 12;13:2638. doi: 10.1038/s41467-022-30303-w (PMC9098523; doi:10.1038/s41467-022-30303-w)
Supplement: Supplementary file 2 — Description of Additional Supplementary Information [file 41467_2022_30303_MOESM2_ESM.pdf]

Description of Additional Supplementary Files

Supplementary Movies:

Supplementary movie 1: Three-dimensional image of MRNIP-GFP droplets in cells. Cells expressing MRNIP-GFP were observed with a confocal microscope using Z-stack mode.

Supplementary movie 2: Fusion of two adjacent MRNIP-GFP droplets in cells. Cells expressing MRNIP-GFP were observed with a confocal microscope using time series mode.

Supplementary movie 3: Fission of MRNIP-GFP droplet in cells. Long-time observation of cells expressing MRNIP-GFP showed that one MRNIP-GFP droplet could fissure to two smaller ones.

Supplementary movie 4: MRNIP droplets float and settle onto coverslip *in vitro*. Recombinant MRNIP-GFP protein was diluted in buffer containing 20 mM Tris-HCl, pH7.4 and 150 mM NaCl to a final concentration of 10 μM. The solution was observed with a confocal microscope.

Supplementary movie 5: Fusion of MRNIP-GFP droplets *in vitro*. Fusion of adjacent MRNIP-GFP droplets was observed in solution that analyzed in Movie 4.

Supplementary movie 6: The dynamic changes of chromatin condensates
