# Supplementary material for: MRNIP condensates promote DNA double-strand break sensing and end resection
Source: Nat Commun. 2022 May 12;13:2638. doi: 10.1038/s41467-022-30303-w (PMC9098523; doi:10.1038/s41467-022-30303-w)
Supplement: Supplementary file 9 — Reporting Summary [file 41467_2022_30303_MOESM9_ESM.pdf]

nature portfolio

Corresponding author(s): Mien-Chie Hung

Last updated by author(s): Apr 11, 2022

Reporting Summary

Nature Portfolio wishes to improve the reproducibility of the work that we publish. This form provides structure for consistency and transparency in reporting. For further information on Nature Portfolio policies, see our Editorial Policies and the Editorial Policy Checklist.

Statistics

For all statistical analyses, confirm that the following items are present in the figure legend, table legend, main text, or Methods section.

n/a	Confirmed	
☐	☒	The exact sample size (*n*) for each experimental group/condition, given as a discrete number and unit of measurement
☐	☒	A statement on whether measurements were taken from distinct samples or whether the same sample was measured repeatedly
☐	☒	The statistical test(s) used AND whether they are one- or two-sided<br>*Only common tests should be described solely by name; describe more complex techniques in the Methods section.*
☐	☒	A description of all covariates tested
☐	☒	A description of any assumptions or corrections, such as tests of normality and adjustment for multiple comparisons
☐	☒	A full description of the statistical parameters including central tendency (e.g. means) or other basic estimates (e.g. regression coefficient) AND variation (e.g. standard deviation) or associated estimates of uncertainty (e.g. confidence intervals)
☐	☒	For null hypothesis testing, the test statistic (e.g. *F*, *t*, *r*) with confidence intervals, effect sizes, degrees of freedom and *P* value noted<br>*Give P values as exact values whenever suitable.*
☒	☐	For Bayesian analysis, information on the choice of priors and Markov chain Monte Carlo settings
☒	☐	For hierarchical and complex designs, identification of the appropriate level for tests and full reporting of outcomes
☒	☐	Estimates of effect sizes (e.g. Cohen's *d*, Pearson's *r*), indicating how they were calculated

Our web collection on statistics for biologists contains articles on many of the points above.

Software and code

Policy information about availability of computer code

Data collection	Bio-rad ChemiDoc Imaging System<br>ZEISS LSM 880<br>icon scanner AFM instrument (Dimension FastScan Bio)<br>NanoScope Analysis software (Version 1.40)
Data analysis	The software used to analyze data in this study are as follow: GraphPad Prism version 8, SPSS version 13.0, ZEN blue edition version 3.1, Image Lab version 5.1, ImageJ version 1.8.0, NanoScope Analysis v300r1, FlowJo X 10.0.7.

For manuscripts utilizing custom algorithms or software that are central to the research but not yet described in published literature, software must be made available to editors and reviewers. We strongly encourage code deposition in a community repository (e.g. GitHub). See the Nature Portfolio guidelines for submitting code & software for further information.

Data

Policy information about availability of data

All manuscripts must include a data availability statement. This statement should provide the following information, where applicable:

- Accession codes, unique identifiers, or web links for publicly available datasets
- A description of any restrictions on data availability
- For clinical datasets or third party data, please ensure that the statement adheres to our policy

Data are available in the main figures and Supplementary Figures. The source data underlying Figs. 1e, 1f-j, 2a-b, 2k, 2n, 3d, 4a, 4c, 4e, 4g, 5a, 5c-i, 6b-i, 7a-g, 7i-k, and Supplementary Figs. 2a-f, 3f-j, 4f, 4i-l, 5f, 5h, 6a, 6c, 6g-i, 7a-m, 8b-j and 9 are provided as a Source Data file. Source data are provided with this paper.

Field-specific reporting

Please select the one below that is the best fit for your research. If you are not sure, read the appropriate sections before making your selection.

☒ Life sciences ☐ Behavioural & social sciences ☐ Ecological, evolutionary & environmental sciences

For a reference copy of the document with all sections, see nature.com/documents/nr-reporting-summary-flat.pdf

Life sciences study design

All studies must disclose on these points even when the disclosure is negative.

Sample size	No sample size calculation was performed. Sample size was determined according to our experience as well as literature reporting in terms of specific experiment. For xenograft mouse models, 4 mice were randomly chose for each group.
Data exclusions	No data were excluded from the analysis.
Replication	Experiments from clinical samples, including Fig. 1d and Supplementary Fig. 1h, were repeated once. Experiments including Fig. 1b-c, 2c-e, 2h, 2m, 3c, 3g-i, 4d-h, 5b-f, 6b-c and Supplementary Fig. 1b-g, 3a, 5a-c, 6a-d, 6f-i, 7b-f, 7i, 7k, 7l, 8a-c, 8k were repeated independently three times. Statistical analyses for each experiment are indicated in the respective figure legends. All replication attempts were successful.
Randomization	The C57BL/6 mice used in xenograft experiments were randomly allocated into IR or control groups.
Blinding	Data measurement for Immunofluorescence, Immunohistochemistry, flow cyto, Flow cytometry and colony formation were blinded to person who performed analysis.

Reporting for specific materials, systems and methods

We require information from authors about some types of materials, experimental systems and methods used in many studies. Here, indicate whether each material, system or method listed is relevant to your study. If you are not sure if a list item applies to your research, read the appropriate section before selecting a response.

Materials & experimental systems

n/a	Involved in the study	
☐	☒	Antibodies
☐	☒	Eukaryotic cell lines
☒	☐	Palaeontology and archaeology
☐	☒	Animals and other organisms
☐	☒	Human research participants
☒	☐	Clinical data
☒	☐	Dual use research of concern

Methods

n/a	Involved in the study	
☒	☐	ChIP-seq
☐	☒	Flow cytometry
☒	☐	MRI-based neuroimaging

Antibodies

Antibodies used	The primary antibodies used in Western blot targeted the following proteins: MRE11 (4847S, CST, 1:1000), Rad50 (3427S, CST, 1:1000), NBS1 (14956S, CST, 1:1000), p-NBS1 (3001S, CST, 1:1000), MRNIP (ab150917, Abcam, Cambridge, MA, USA, 1:1000), Lamin A/C (ab108595, Abcam, 1:4000), p-H2A.X (9718S, CST, 1:1000), GAPDH (60004-1-Ig, Proteintech, Rosemont, IL, USA, 1:5000), p-ATM-S1981 (AP0008, Abclonal, Wuhan, China, 1:1000), p-CHK1-S345 (2348S, CST, 1:1000), α-Tubulin (66031-1-Ig, Proteintech, 1:5000) and H2A.X (A11361, Abclonal, 1:1000).<br>The primary antibodies used in Immunofluorescence targeted the following proteins: MRNIP (ab157629, Abcam, 1:200; ab150917, Abcam, 1:200; TA330650, Origene, 1:200), MRE11 (ab214, Abcam, 1:100), p-H2A.X (9718S, CST, 1:500), p-H2A.X (80312S, CST, 1:500), p-ATM-S1891 (AP0008, Abclonal, 1:300), RPA1 (2267S, CST, 1:50), TP53BP1 (4937, CST, 1:200), Rad51 (ET1705-96, HuaBio, Hangzhou, China, 1:100) and BrdU (66241-1-Ig, Proteintech, 1:300).<br>The primary antibodies used in Immunohistochemistry targeted the following proteins: MRNIP (ab157629, Abcam, 1:100; TA330650, Origene, 1:100), Ki67 (ET1609-34, HuaAn biotechnology, Hangzhou, China, 1:200).
Validation	All antibodies used in this study were validated for their applications by the manufacturers or by ourselves using genetic knockouts.<br>anti-MRE11 (4847S, CST): https://www.cellsignal.com/products/primary-antibodies/mre11-31h4-rabbit-mab/4847?site-search-type=Products&N=4294956287&Ntt=4847s&fromPage=plp&_requestid=3574349<br>anti-Rad50 (3427S, CST): https://www.cellsignal.com/products/primary-antibodies/rad50-antibody/3427?site-search-type=Products&N=4294956287&Ntt=3427s%5C&fromPage=plp&_requestid=3574396<br>anti-NBS1 (14956S, CST): https://www.cellsignal.com/products/primary-antibodies/p95-nbs1-d6j5i-rabbit-mab/14956?site-search-type=Products&N=4294956287&Ntt=14956s&fromPage=plp&_requestid=3574423<br>anti-p-NBS1 (3001S, CST): https://www.cellsignal.com/products/primary-antibodies/phospho-p95-nbs1-ser343-antibody/3001?site-search-type=Products&N=4294956287&Ntt=3001s&fromPage=plp&_requestid=3574447

March 2021

anti-MRNIP (ab150917, Abcam): https://www.abcam.com/c5orf45-antibody-ab150917.html
anti-Lamin A/C (ab108595,Abcam): https://www.abcam.com/lamin-a--lamin-c-antibody-epr4100-nuclear-envelope-marker-ab108595.html
anti-p-H2A.X (9718S, CST): https://www.cellsignal.com/products/primary-antibodies/phospho-histone-h2a-x-ser139-20e3-rabbit-mab/9718?site-search-type=Products&N=4294956287&Ntt=+%289718s&fromPage=plp&_requestid=3574709
anti-GAPDH (60004-1-Ig, Proteintech): https://www.ptglab.com/products/GAPDH-Antibody-60004-1-Ig.htm
anti-p-ATM-S1981 (AP0008, Abclonal): https://abclonal.com.cn/catalog/AP0008
anti-p-CHK1-S345 (2348S, CST): https://www.cellsignal.com/products/primary-antibodies/phospho-chk1-ser345-133d3-rabbit-mab/2348?site-search-type=Products&N=4294956287&Ntt=2348s&fromPage=plp&_requestid=3575775
anti-α-Tubulin (66031-1-Ig, Proteintech): https://www.ptglab.com/products/tubulin-Alpha-Antibody-66031-1-Ig.htm
anti-H2A.X (A11361, Abclonal): https://abclonal.com.cn/Datasheet/Antibodies/A11361.pdf
anti-MRNIP (157629, Abcam): https://www.abcam.com/c5orf45-antibody-n-terminal-ab157629.html
anti-MRNIP (TA330650, Origene): https://www.origene.com/catalog/antibodies/primary-antibodies/ta330650/c5orf45-mrnip-rabbit-polyclonal-antibody
anti-MRE11 (ab214, Abcam): https://www.abcam.com/mre11-antibody-12d7-bsa-and-azide-free-ab214.html
anti-p-H2A.X (80312S, CST): https://www.cellsignal.com/products/primary-antibodies/phospho-histone-h2a-x-ser139-d7t2v-mouse-mab/80312?_=1649600780989&Ntt=p-H2A.X%20(80312S&tahead=true
anti-RPA1 (2267S, CST): https://www.cellsignal.com/products/primary-antibodies/rpa70-rpa1-antibody/2267?_=1649601470643&Ntt=rpa1%20(2267s,&tahead=true
anti-TP53BP1 (4937, CST): https://www.cellsignal.com/products/primary-antibodies/53bp1-antibody/4937?site-search-type=Products&N=4294956287&Ntt=tp53bp1+%284937&fromPage=plp&_requestid=3577300
anti-Rad51 (ET1705-96, HuaBio): https://www.huabio.com/products/rad51-antibody-clone-jm54-26-recombinant-monoclonal-et1705-96
anti-BrdU (66241-1-Ig, Proteintech): https://www.ptglab.com/products/BrDu-Antibody-66241-1-Ig.htm
anti-Ki67 (ET1609-34, HuaAn biotechnology): https://www.huabio.com/products/ki67-antibody-clone-st50-01-recombinant-monoclonal-et1609-34

Eukaryotic cell lines

Policy information about cell lines

Cell line source(s)	HEK 293T cells (ATCC), HeLa cells (Guangzhou Cellcook Biotech Co., Ltd), sf9 insect cells (Guangzhou Cellcook Biotech Co., Ltd).
Authentication	HEK 293T, HeLa and sf9 cells were authenticated using STR profiling by the provider ATCC and Guangzhou Cellcook Biotech Co., Ltd.
Mycoplasma contamination	All cell lines were mycoplasma-free.
Commonly misidentified lines (See ICLAC register)	No commonly misidentified cell lines were used.

Animals and other organisms

Policy information about studies involving animals; ARRIVE guidelines recommended for reporting animal research

Laboratory animals	5-week-old male NOG mice (NOD.Cg-PrkdcscidIl2rgtm1Sug/JicCrl) purchased from Charles River were used in this study. All mice were housed under specific pathogen-free conditions with access to food and water. All mice were maintained in ambient room temperature (23 +/- 3?) with humidity of 40-70% and light/dark cycle of 12h/12h.
Wild animals	No wild animals were used in this study.
Field-collected samples	No field-collected samples were used in this study.
Ethics oversight	All animal experiments were approved by the Institutional Animal Care and Use Committee of the Sixth Affiliated Hospital of Sun Yat-sen University (Accreditation No. IACUC-2020052503).

Note that full information on the approval of the study protocol must also be provided in the manuscript.

Human research participants

Policy information about studies involving human research participants

Population characteristics	tissues from CRC patients: CRC and adjacent non-tumor tissues from 213 patients. All these patients were Chinese. Their mean age was 54.08; Male vs Female=150 vs 63. More details could be found in Source Data.
Recruitment	Tissues were obtained from the Tissue Bank of the Sixth Affiliated Hospital, Sun Yat-sen University. CRC Patients receiving neo-adjuvant chemo-radiotherapy and subsequent radical CRC surgery among 2010-2017 were involved.

Ethics oversight	Informed consent was obtained from each patient, and the protocol was approved by the Institutional Research Ethics Committee of the Sixth Affiliated Hospital of Sun Yat-sen University.

Note that full information on the approval of the study protocol must also be provided in the manuscript.

Flow Cytometry

Plots

Confirm that:

☒ The axis labels state the marker and fluorochrome used (e.g. CD4-FITC).

☒ The axis scales are clearly visible. Include numbers along axes only for bottom left plot of group (a 'group' is an analysis of identical markers).

☒ All plots are contour plots with outliers or pseudocolor plots.

☒ A numerical value for number of cells or percentage (with statistics) is provided.

Methodology

Sample preparation	For DNA repair reporter analysis, cells were seeded in 10cm dish for 24 hours before transfected with 2.5 ug pLCN DSB Repair Reporter, 4 ug pCAGGS DRR mCherry Donor EF1a BFP and 2.5 ug pCBASceI plasmid (these plasmids were gifts from Jan Karlseder (Addgene plasmid # 98896; http://n2t.net/addgene:98896; RRID: Addgene_98896). Forty-eight hours after transfection, cells were trypsinized and resuspended in 1×PBS supplemented with 5% FBS and subjected to flow cytometry analysis. For cell cycle analysis, cells were trypsinized, resuspended in a detergent-containing hypotonic solution and subjected to flow cytometry analysis as described previously.
Instrument	CytoFLEX, Beckman, Pasadena, USA
Software	FlowJo X 10.0.7 was used to analyze data.
Cell population abundance	Cell line were used in this experiments, which was a pure population.
Gating strategy	BFP+ cells were gated for mCherry analysis.

☒ Tick this box to confirm that a figure exemplifying the gating strategy is provided in the Supplementary Information.
